# A Highly Expressed Odorant Receptor Detects the Aggregation Pheromone Rhynchophorol in the Invasive American Palm Weevil

**DOI:** 10.1111/mec.70504

**Published:** 2026-08-09

**Authors:** Sai Zhang, Nicolas Montagné, Binu Antony, Guirong Wang, Mark Hoddle, Arnab Pain, Emmanuelle Jacquin‐Joly

**Affiliations:** ^1^ INRAE, Sorbonne Université, CNRS, IRD, UPEC, Université Paris Cité, Institute of Ecology & Environmental Sciences of Paris Versailles Cedex France; ^2^ Shenzhen Branch, Guangdong Laboratory for Lingnan Modern Agriculture, Key Laboratory of Synthetic Biology, Ministry of Agriculture and Rural Affairs, Agricultural Genomics Institute at Shenzhen Chinese Academy of Agricultural Sciences Shenzhen China; ^3^ Institut Universitaire de France Paris France; ^4^ Khalifa Center for Genetic Engineering and Biotechnology United Arab Emirates University (UAEU) Al Ain Abu Dhabi UAE; ^5^ State Key Laboratory for Biology of Plant Diseases and Insect Pests, Institute of Plant Protection Chinese Academy of Agricultural Sciences Beijing China; ^6^ Department of Entomology University of California Riverside California USA; ^7^ Bioscience Programme, BESE Division King Abdullah University of Science and Technology (KAUST) Thuwal, Jeddah Saudi Arabia

**Keywords:** aggregation pheromone, pheromone receptor, *Rhynchophorus palmarum*, *Xenopus* oocyte

## Abstract

Communication via aggregation pheromones is responsible for the behavioural and ecological characteristics of many species of weevils (Coleoptera). In insects, pheromones are detected by specialized odorant receptors (ORs), called pheromone receptors (PRs), which are usually highly expressed in olfactory sensory neurons localized in the antennae. Yet, PRs in Coleoptera remain understudied, which limits our understanding of their response characteristics and potential multiple evolutionary origins. In this study, we search for PRs in the American palm weevil, *Rhynchophorus palmarum* , a pest species that poses a major threat to oil palm and coconut production in the Americas, for which no PR responding to its aggregation pheromone (2E)‐6‐methyl‐2‐hepten‐4‐ol (rhynchophorol) has been characterized. Combining published RNA‐seq data with new data generated in this study, we identified two ORs highly expressed in the weevil antennae that represent strong candidate aggregation PRs. Sequence‐based binding pocket prediction revealed overall structural similarity between these two ORs, but amino acid differences were identified, suggesting functional divergence. Using the *Xenopus* oocyte heterologous expression system, we demonstrated that RpalOR32 displayed specificity and strong sensitivity to the aggregation pheromone rhynchophorol, with minor responses to several structurally related compounds, while RpalOR25 showed no responses to pheromones or host plant volatiles. The newly identified PR in *R. palmarum* appeared phylogenetically distant from the aggregation PR previously identified in the related species *R. ferrugineus* , confirming the independent origin of weevil PRs, even at the genus level.

## 1 Introduction

Insects are surrounded by a variety of volatile chemicals that play crucial roles in their behaviour and ecology. Behaviours guided by chemical cues include mate location, oviposition site and food source selection, and predator detection (Andersson et al. 2015; Bruce et al. 2005; Fleischer et al. 2018; Hansson and Stensmyr 2011). Among these chemicals, pheromones, such as sex or aggregation pheromones, are key intraspecific communication signals in insects. The detection of pheromones is ensured by odorant receptors (ORs), which are expressed in the dendritic membranes of olfactory sensory neurons (OSNs) housed in antennal olfactory sensilla (de Bruyne and Baker 2008). Insect ORs are atypical seven‐transmembrane proteins with an inverted topology compared to G‐protein coupled receptors (Benton et al. 2006; Lundin et al. 2007). Insect ORs function as heteromeric ion channels in combination with a highly conserved co‐receptor, Orco (Sato et al. 2008; Wicher et al. 2008). The OR/Orco complexes assemble as heterotetramers, typically in a 3 Orco:1 tuning OR stoichiometry (Wang et al. 2024; Zhao et al. 2024). Within this architecture, ligand recognition is exclusively mediated by the variable OR subunit, while Orco serves as a conserved structural scaffold that stabilizes the complex and ensures channel function. Ligand‐binding elicits conformational changes in the OR subunit, particularly an outward shift of the S7b helix, resulting in an asymmetric opening of the central ion‐conducting pore. In contrast, the Orco subunits remain largely unchanged between ligand‐free and ligand‐bound states (Wang et al. 2024; Zhao et al. 2024).

The insect OR gene family is known to diversify quickly, following a classical ‘birth‐and‐death’ evolutionary pattern (Robertson 2019). This dynamic process leads to lineage‐specific expansions or losses of OR genes and contributes to substantial sequence divergence across taxa. As a result, clear OR orthology is typically restricted to closely related species within the same insect family. Insect ORs display a wide range of tuning properties: some receptors are narrowly selective and detect only one or a few structurally similar odorants, while others respond to a broader spectrum of chemicals (de Fouchier et al. 2017; Hallem and Carlson 2006; Wang et al. 2010). In particular, ORs tuned to pheromones, also called pheromone receptors (PRs), tend to evolve strong ligand specificity, enabling precise detection of intraspecific signals, as demonstrated by numerous studies focusing on Lepidoptera sex PRs. In moths, most PRs cluster into a few well‐defined phylogenetic groups, and one of these groups includes the majority of PRs. This distribution points to an ancient origin for PRs involved in mate communication. In contrast, current understanding of PR evolution in Coleoptera remains limited, despite Coleoptera being a major insect order with enormous species diversity. Only a handful of coleopteran receptors have been characterized, and PRs tuned to conspecific or heterospecific (anti‐)aggregation pheromones have only been reported in eight beetle species, mostly Curculionidae. These include the two destructive palm weevils *Rhynchophorus ferrugineus* and *R. palmarum* (Antony et al. 2021; Brajon et al. 2024), the spruce bark beetle, *Ips typographus* (Biswas, Sims, et al. 2024; Hou et al. 2021; Johny et al. 2025; Yuvaraj et al. 2024, 2021), the ambrosia beetles, *Trypodendron lineatum* and *T. domesticum* (Andersson et al. 2025), the scarab beetles, *Anomala corpulenta* and *Popillia japonica* (Wang et al. 2026), and the cerambycid beetle, *Megacyllene caryae* (Mitchell et al. 2012). Current data do not support the presence of a large PR lineage in Coleoptera. Instead, beetle PRs are dispersed across the OR phylogeny. For instance, although all known Curculionidae PRs fall within the major coleopteran OR subfamily known as Group 7—one of the nine main ancestral OR lineages in beetles—they are distributed among several distinct sub‐clades (Andersson et al. 2025). This pattern indicates that PRs likely originated multiple times independently in Curculionidae. Additionally, Group 7 includes numerous ORs that respond to non‐pheromonal odorants (Hou et al. 2021), highlighting its functional diversity beyond pheromone detection. Identifying additional PRs in Coleoptera is thus essential for elucidating their evolutionary trajectories and ligand specificities.

Recently, we identified conserved pheromone receptor orthologues in two weevil species, the Asian palm weevil, *R. ferrugineus* , and the American palm weevil, *R. palmarum* , both of which are recognized as devastating pests of palms (Arecaceae) (Antony et al. 2021; Brajon et al. 2024). Both species rely on distinct aggregation pheromones released by males, which attract conspecific males and females for feeding, mating, and ovipositing (Hallett et al. 1993; Rochat et al. 1991). As an example of clear OR orthology in related species, OR1 in these two species is activated by the two components of the aggregation pheromone of *R. ferrugineus* , ferrugineol (4‐methyl‐5‐nonanol) and ferrugineone (4‐methyl‐5‐nonanone), and to structurally related compounds (Antony et al. 2021; Brajon et al. 2024). However, the ecological function of OR1 differs between the two species. While it is involved in intraspecific communication in *R. ferrugineus* , it is not in *R. palmarum* , whose aggregation pheromone consists of (2E)‐6‐methyl‐2‐hepten‐4‐ol (rhynchophorol) (Hallett et al. 1993; Rochat et al. 1991) and is not detected by OR1 (Brajon et al. 2024). Yet, the *R. palmarum* PR tuned to rhynchophorol remains unknown. Therefore, the aim of this study was to identify the PR for rhynchophorol in *R. palmarum* and clarify the evolutionary trajectories of PRs in Coleoptera.

A previous study identified 63 ORs and one Orco in the antennal transcriptome of *R. palmarum* (Gonzalez et al. 2021). However, partial sequence annotations for most ORs hinder functional studies. Here, we first generated new RNA‐seq data from *R. palmarum* antenna to complete OR sequences. *Rhynchophorus palmarum* ORs clustered across six of the nine major coleopteran OR groups defined to date, with the majority belonging to groups 1, 2, 5, and 7 (Gonzalez et al. 2021; Mitchell et al. 2020). With the hypothesis that the *R. palmarum* OR(s) (further referred to as RpalORs) tuned to rhynchophorol should be located within Group 7 and exhibit high expression levels (as observed for other weevil aggregation PRs, Antony et al. 2021), we selected the two most highly expressed RpalORs in both male and female antennae located in Group 7, namely RpalOR32 and RpalOR25, for subsequent functional studies. Using the *Xenopus laevis* oocyte heterologous expression system coupled with two‐electrode voltage clamp, we revealed that RpalOR32 showed selective, highly sensitive responses to the aggregation pheromone, rhynchophorol. Notably, RpalOR32 did not respond to any of the plant volatiles tested, contrary to observations for RferOR1 (Brajon et al. 2024). In contrast, RpalOR25 showed no detectable responses to 40 tested compounds. While RferOR1 and RpalOR32 share the same ecological function (detecting their respective conspecific aggregation pheromone), they are localized in distinct OR lineages within Group 7. Moreover, RpalOR32 clustered with a recently identified PR in *Ips typographus*, also a curculionid (Johny et al. 2025), defining a potential new PR subfamily. Altogether, these data suggest multiple origins of aggregation PRs, even in related species from the same genus, within the Curculionidae.

## 2 Materials and Methods

### 2.1 *Rhynchophorus palmarum* Collection and Antennal Tissue Dissection

Male and female adults were field‐collected in pheromone‐baited traps at the Sweetwater Reserve, Bonita San Diego County, California USA. Weevils used for analyses were captured over the period October 2021 to March 2022. All live weevils were transferred to clean ventilated plastic containers, fed cut pieces of apple and banana, and moved to the University of California Insectary and Quarantine Facility under California Department of Food and Agriculture permit 3268. Once in the laboratory, the insects were sexed and placed in a freezer (−8°C) for about 10 min for immobilization and anaesthesia. Antennae from 30 males and 25 females were excised using fine entomological scissors cleaned with 95% ethanol. Excised antennae were stored in labelled Eppendorf tubes containing RNAlater (Thermo‐Fisher Scientific, WA, USA) for later analysis.

### 2.2 Antennal Transcriptome and OR Annotation

Total RNAs were extracted from 10 pairs of antennae from either male or female *R. palmarum* using the PureLink RNA Mini Kit (Invitrogen, USA), according to the manufacturer's instructions. The quantity and quality of the total RNAs were validated using a Qubit 2.0 Fluorometer (Invitrogen, Life Technologies), and RNA integrity was further confirmed using a 2100 Bioanalyzer (Agilent Technologies). Paired‐end cDNA libraries were prepared using the TruSeq Stranded mRNA Library Prep Kit (Illumina Inc.) according to the manufacturer's protocols. Illumina HiSeq 2000 sequencing was performed at KAUST's core sequencing facility in Saudi Arabia. Image deconvolution and quality value calculations were performed using Illumina's GAPipeline 1.3. Illumina adaptors were detected and removed by an automatic read‐through adapter trimming option implemented in the ‘Trim Reads’ tool of Qiagen CLC Genomics Server (CLC) (v 21.0.1). Low‐quality bases were also trimmed off, allowing two ambiguities per read. Filtered paired‐end reads were QC validated through a ‘QC for Sequencing Reads Tool’ of CLC. A de novo transcriptome assembly was constructed with the ‘De Novo Assembly Tool’ of CLC, and OR transcripts were identified using BLASTN and *R. palmarum* ORs previously described as queries (Gonzalez et al. 2021).

### 2.3 Phylogenetic Analysis of Coleoptera ORs

**FIGURE 1:**
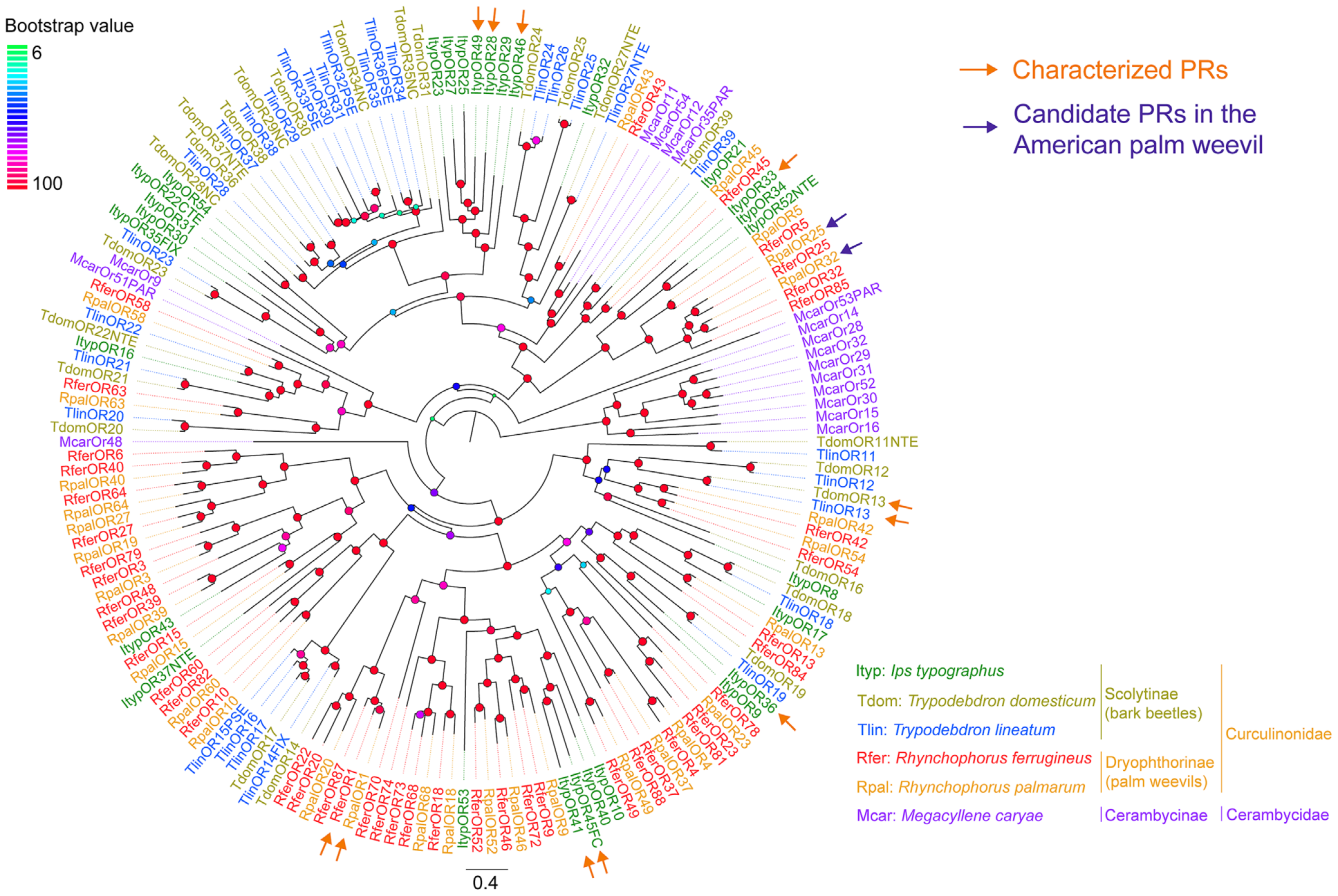
Maximum likelihood tree of the Group 7 of coleopteran odorant receptors (ORs). The phylogenetic tree was constructed from OR amino acid sequences of *Ips typographus* (Ityp: Green), *Trypodendron domesticum* (Tdom: Olive green), *Trypodendron lineatum* (Tlin: Blue), *Rhynchophorus ferrugineus* (Rfer: Red), *Rhynchophorus palmarum* (Rpal: Orange), and *Megacyllene caryae* (Mcar: Purple) using IQ‐TREE v2.3.6, and applying the maximum‐likelihood (ML) method with the best‐fitting substitution model (JTT + F + R8) selected by ModelFinder based on the Bayesian Information Criterion (BIC). Previously characterized ORs that respond to pheromones are indicated by orange arrows, and candidate *R. palmarum* PRs investigated in this study are indicated by purple arrows.

A phylogenetic tree was constructed based on the OR sequences from six beetle species in which PRs have been functionally identified: *R. ferrugineus* (Antony et al. 2016) and *R. palmarum* (Curculionidae: Dryophthorinae) (Gonzalez et al. 2021), *I. typographus* (Yuvaraj et al. 2021), *T. domesticum* (Andersson et al. 2025), and *T. lineatum* (Curculionidae: Scolytinae) (Biswas, Vogel, et al. 2024), and *M. caryae* (Cerambycidae: Cerambycinae) (Mitchell et al. 2012). Amino acid sequences were aligned using MAFFT v7 with default parameters (https://mafft.cbrc.jp/). The phylogeny was constructed using IQ‐TREE v2.3.6, applying the maximum‐likelihood (ML) method with the best‐fitting substitution model (JTT + F + R8) selected by ModelFinder based on the Bayesian Information Criterion (BIC) (Kalyaanamoorthy et al. 2017; Nguyen et al. 2015). Node support was assessed with 1000 ultrafast bootstrap (UFBoot) replicates (Hoang et al. 2018). Following the construction of the OR phylogeny, the tree was rooted using the Orco clade (Appendix S1). Then, Group 7, which includes all characterized Curculionidae PRs, was isolated and is displayed in Figure 1. The tree was visualized using FigTree 1.4.0 (http://tree.bio.ed.ac.uk/software/figtree/).

### 2.4 Structural Modelling and Binding Pocket Comparison Between the Two *R. palmarum* Candidate PRs

The full‐length open reading frames (ORFs) of *RpalOR25*, *RpalOR32*, and *RpalOrco* were retrieved from the sequenced antennal transcriptome. Amino‐acid sequence alignments of RpalOR25 and RpalOR32 were performed with DNAMAN v8 (Lynnon Biosoft, Quebec, Canada). For each OR, five structural models were generated with AlphaFold3 (Josh et al. 2024), and the model with the highest pLDDT score was used for binding pocket prediction by PrankWeb (https://prankweb.cz/). The selected 3D structures of RpalOR32 and RpalOR25 were structurally aligned in PyMOL v3.1.6.1 (DeLano 2002) with published cryo‐EM structures of ligand‐bound ORs ( *Acyrthosiphon pisum* ApisOR5–geranyl acetate, *Aedes aegypti* AaegOR10–o‐cresol, and *Anopheles gambiae* AgamOR28–2,4,5‐trimethylthiazole). Protein Data Bank accessions: 8Z9A, 8V02, 8V3D to infer the putative ligand‐binding pocket and enable structural comparison between two candidate PRs. Residues positioned within 5 Å of the predicted pocket center were considered as part of the binding site. The transmembrane domain topology of each receptor was predicted using the PPM web server (https://opm.phar.umich.edu/ppm_server).

### 2.5 Functional Characterization in *Xenopus* Oocytes

The recombinant pCS2+ plasmids containing the optimized sequences of *RpalOR25*, *RpalOR32*, and *RpalOrco* for *Xenopus* expression (sequences available in Appendix S2) were synthesized by Synbio Technologies (New Jersey, USA) and linearized using NotI (ThermoFisher Scientific, Pittsburgh, PA, USA). The linearized templates were purified and transcribed into capped RNA (cRNA) using the mMESSAGE mMACHINE SP6 kit (ThermoFisher Scientific, Pittsburgh, PA, USA). Oocytes were purchased from EcoCyte Bioscience (Dortmund, Germany) and were microinjected with 27.6 nL of a cRNA mixture (RpalORx: RpalOrco 1: 1, 1 ng/nL each) or 27.6 nL of RNase‐free water (control oocytes). After injection, oocytes were incubated in 1 × Ringer's buffer (96 mM NaCl, 2 mM KCl, 5 mM MgCl_2_, 0.8 mM CaCl_2_, and 5 mM HEPES, pH 7.6) supplemented with 5% dialyzed horse serum, 50 mg/mL tetracycline, 100 mg/mL streptomycin, and 550 mg/mL sodium pyruvate. After 3–4 days of incubation at 18°C, the whole‐cell currents of each injected oocyte (*n* = 6–9, depending on the experiments) with a holding potential of −80 mV were recorded using two‐electrode voltage clamp (TURBO‐TEC‐03X, npi electronic GmbH, Tamm, Germany). During the recording, oocytes were exposed to different chemical compound solutions with an interval between exposures that allowed the current to return to baseline. Data acquisition and analysis were performed using the Digidata 1550 and pCLAMP 10.6 software (Axon Instruments Inc., Union City, CA, USA). The tested oocytes were exposed to chemical compound solutions diluted at different concentrations. Differences in current responses between rhynchophorol and each other tested compounds were analysed using paired *t*‐tests, followed by Holm correction for multiple comparisons.

### 2.6 Chemical Compounds

A total of 40 compounds, including the palm weevil aggregation pheromone compounds (ferrugineol: 4‐methyl‐5‐nonanol, ferrugineone: 4‐methyl‐5‐nonanone, rhynchophorol: (E)‐6‐methyl‐2‐hepten‐4‐ol), structurally related compounds, and plant volatile organic compounds (VOCs), were used to test the response spectra of the two ORs. All these compounds were the same as previously used for the functional characterization of OR1 from *R. ferrugineus* and *R. palmarum* (Brajon et al. 2024) and are listed in the Appendix S3. Compounds were dissolved to 1 M in dimethyl sulfoxide (DMSO) to form stock solutions, which were stored at −20°C. Before experiments, the stock solutions were diluted in 1 × Ringer's buffer to working concentrations of 10^−4^ M and 10^−5^ M. All compounds were tested at 10^−4^ M on RpalOR25, a dilution previously used for *Rhynchophorus* OR characterization (Ji et al. 2021). For RpalOR32, plant volatiles were also tested at 10^−4^ M, whereas aggregation pheromones and their structurally related compounds were tested at 10^−5^ M, to prevent potential damage to the oocyte membrane caused by excessive responses to rhynchophorol. For dose–response recordings, a 10^−9^ to 3 × 10^−5^ M concentration range of rhynchophorol was used. 1 × Ringer's buffer containing 0.1% DMSO was used as a negative control.

## 3 Results

### 3.1 *Rhynchophorus palmarum* OR Annotation

RNA‐seq data have been uploaded to NCBI under BioProject PRJNA656150 and SRA accession numbers: SRR29255395 (male antennae) and SRR29255396 (female antennae). The 63 *R. palmarum* ORs and Orco previously described (Gonzalez et al. 2021) were all identified in the newly assembled transcriptome, and 2 sequences could be completed, leading to a total of 41 complete *R. palmarum* OR sequences (Appendix S4). Importantly, the complete sequence of RpalOR32 that was partial in previous data (Gonzalez et al. 2021) could be obtained. No additional OR was annotated.

### 3.2 Selection of Candidate *Rhynchophorus palmarum* PRs

To identify the aggregation PR(s) of *R. palmarum* , we targeted two ORs, RpalOR32 and RpalOR25, exhibiting the highest antennal expression levels among RpalORs, representing 32.1% and 31.8% of Orco expression, respectively (Gonzalez et al. 2021). Phylogenetic analyses further showed that these two ORs were located in Group 7 (Figure 1), which includes all currently characterized curculionid PRs. Additionally, RpalOR32 and RpalOR25 were adjacent in the phylogenetic tree, suggesting they are potential paralogous genes (Figure 1). They formed a subclade with ItypOR33 from *I*. *typographus*, a pheromone receptor that detects a heterospecific aggregation pheromone from *I*. *amitinus* and few other *Ips* species (Johny et al. 2025). However, these two ORs were phylogenetically distant from other characterized PRs tuned to conspecific aggregation pheromones, including RferOR1 from the closely related red palm weevil *R. ferrugineus* , ItypOR45 and ItypOR41 from *I*. *typographus*, and TdomOR13 and TlinOR13 from *Trypodendron* ambrosia beetles (Figure 1). They were also positioned on distinct branches from other PRs of *I*. *typographus* tuned to conspecific anti‐aggregation pheromones or heterospecific aggregation pheromones, as well as from the heterospecific aggregation pheromone receptor (RpalOR1) of *R. palmarum* (Figure 1).

### 3.3 Sequence and Structural Analysis of the Two Candidate PRs

**FIGURE 2:**
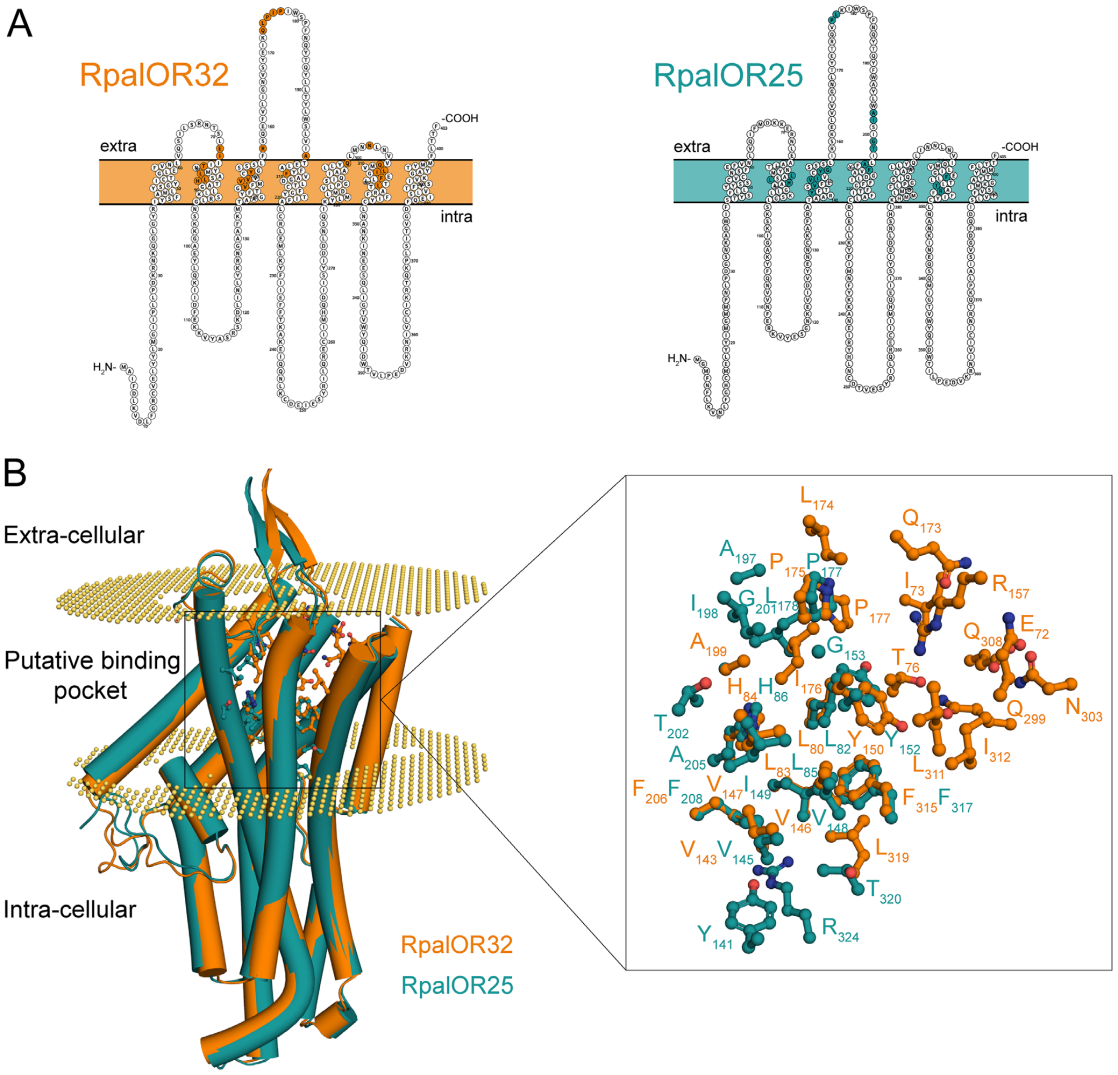
Sequence and predicted structure comparison of RpalOR32 and RpalOR25. (A) Membrane topology of RpalOR32 and RpalOR25, as predicted using the PPM web server (https://opm.phar.umich.edu/ppm_server). Residues predicted to form the binding pockets are highlighted in orange (RpalOR32: E72, I73, T76, L80, L83, H84, V143, V146, V147, Y150, R157, Q173, L174, P175, I176, P177, A199, F206, Q299, N303, Q308, L311, I312, F315, L319) and cyan (RpalOR25: L82, L85, H86, Y141, V145, V148, I149, Y152, G153, P177, L178, A197, I198, G201, T202, A205, F208, F317, T320, R324). The transmembrane regions were plotted using Portter v1.0 (Omasits et al. 2014). (B) Predicted 3D structures of RpalOR32 and RpalOR25, with predicted pockets and transmembrane domains. All residues constructing the binding pocket are displayed as sticks and spheres.

The amino acid sequence identity between RpalOR32 and RpalOR25 was 69.95%, indicating moderate sequence divergence (Figure 2A). Despite this, AlphaFold3‐predicted structures of these two receptors exhibited broadly similar global folds, with an RMSD value of 1.12 Å computed across all amino acids (Figure 2B). Binding pocket prediction identified 25 (E72, I73, T76, L80, L83, H84, V143, V146, V147, Y150, R157, Q173, L174, P175, I176, P177, A199, F206, Q299, N303, Q308, L311, I312, F315, L319) and 20 residues (L82, L85, H86, Y141, V145, V148, I149, Y152, G153, P177, L178, A197, I198, G201, T202, A205, F208, F317, T320, R324) forming the putative ligand‐binding cavities of RpalOR32 and RpalOR25, respectively, which were distributed across transmembrane helices S2‐S6 (Figure 2A). Among these residues, 10 were conserved between the two receptors and were located mainly in the central region of the predicted pockets, whereas receptor‐specific residues were positioned toward the periphery (Figure 2B). This spatial pattern suggests that the two receptors share a conserved core architecture that maintains the overall ligand‐binding framework, while peripheral variations potentially fine‐tune ligand selectivity and contribute to functional differentiation.

### 3.4 RpalOR32, the Most Highly Expressed OR, Is Selective and Sensitive to the Aggregation Pheromone, Rhynchophorol

**FIGURE 3:**
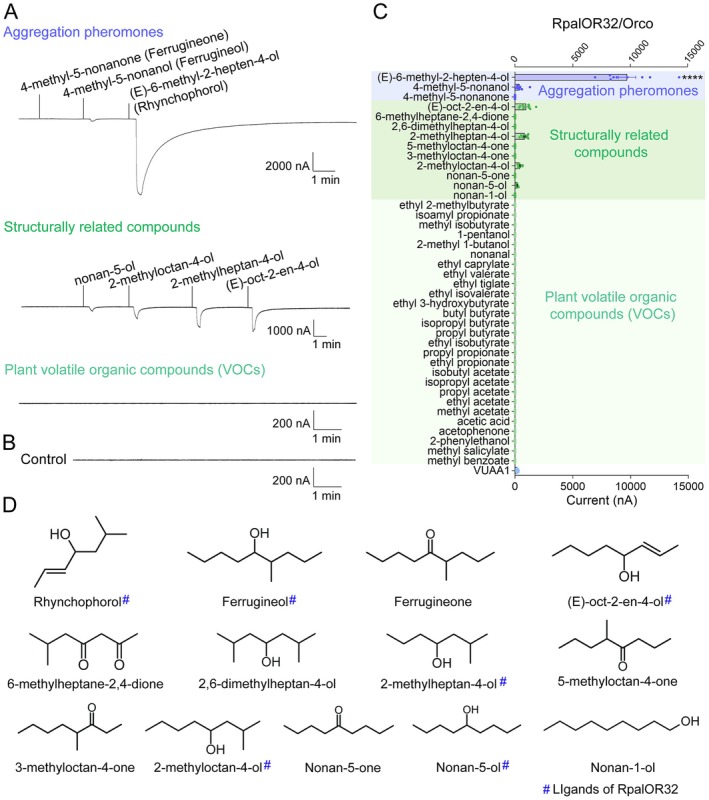
Functional characterization of RpalOR32. (A) Typical response profiles of *Xenopus* oocytes expressing RpalOR32/RpalOrco to aggregation pheromones and structurally related compounds at the concentration of 10^−5^ M, and to plant VOCs at the concentration of 10^−4^ M. (B) Water‐injected control oocytes showed no response to the tested compounds (*n* = 6). (C) Inward current responses of *Xenopus* oocytes expressing RpalOR32/RpalOrco to 10^−5^ M of the aggregation pheromones of *Rhynchophorus palmarum* and *Rhynchophorus ferrugineus* (*n* = 9) (blue shade), to 10^−5^ M of structurally related compounds (*n* = 10) (dark green shade), and to 10^−4^ M plant VOCs (*n* = 6) (light green shade). Error bars indicate SEM. **** adjusted *p* < 0.0001 versus all other tested compounds after Holm correction. (D) Chemical structures of the aggregation pheromones from *R. palmarum* and *R. ferrugineus* and related analogs. Compounds marked with a hashtag sign (#) represent the main and minor ligands of RpalOR32.

To characterize the function of RpalOR32, we co‐expressed RpalOR32 with its co‐receptor RpalOrco in *Xenopus laevis* oocytes and functionally screened a panel of 40 compounds using two‐electrode voltage‐clamp. When the oocytes were exposed to rhynchophorol at 10^−4^ M, extremely strong currents (> 10,000 nA) were elicited. To minimize the potential damage to the oocyte membrane, the concentration was reduced to 10^−5^ M. Even at this lower concentration, RpalOR32 also showed extremely strong responses to rhynchophorol with a mean response of 9704.0 ± 745.3 nA, significantly exceeding those elicited by all other tested compounds (Holm‐adjusted, *p* < 0.0001; Figure 3A,C). In addition, minor responses (< 1000 nA) were elicited by some structurally related compounds, (E)‐oct‐2‐en‐4‐ol, 2‐methylheptan‐4‐ol, 2‐methyloctan‐4‐ol, and nonan‐5‐ol (Figure 3A,C), while the 27 tested VOCs failed to elicit any detectable response (Figure 3A,C). The water‐injected oocytes showed no response to any of the tested compounds (Figure 3B). The ligand profile suggested that RpalOR32 selectively responded to unsaturated or methyl‐substituted alcohols structurally related to rhynchophorol, whereas saturated alcohols and structurally related ketones did not activate this receptor (Figure 3D).

**FIGURE 4:**
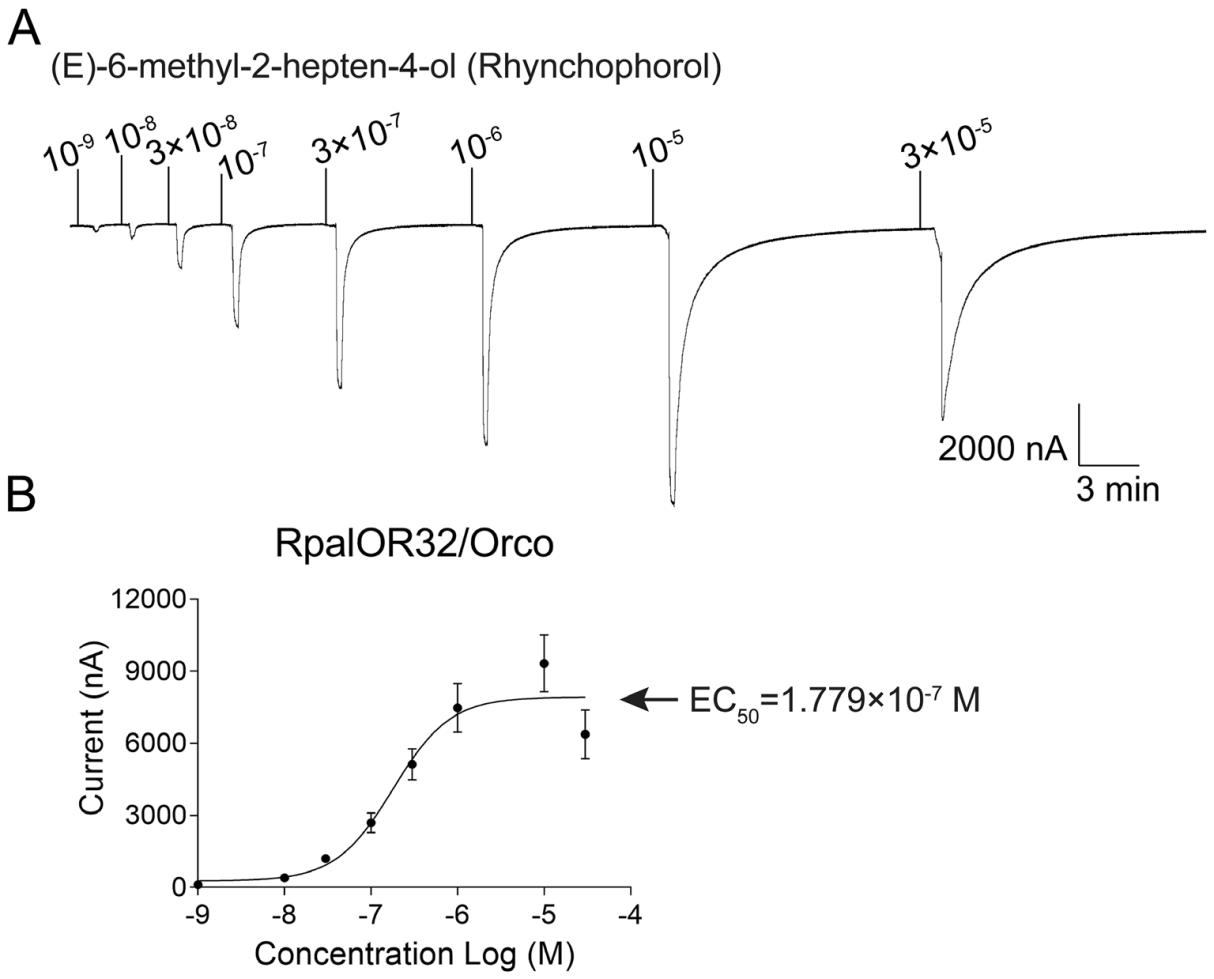
Dose‐dependent responses of *Xenopus* oocytes co‐expressing RpalOR32/RpalOrco to rhynchophorol. (A) Representative current trace of a RpalOR32/RpalOrco *Xenopus* oocyte stimulated with a range of rhynchophorol concentrations. (B) Dose–response curve of RpalOR32/RpalOrco *Xenopus* oocytes to rhynchophorol. Error bars indicate SEM (*n* = 7).

To evaluate the sensitivity of RpalOR32 to rhynchophorol, dose–response recordings were conducted (Figure 4A). The results showed that detectable responses (117.4 ± 20.7 nA) were recorded even at a low concentration of 10^−9^ M, and the response amplitude increased with increasing concentration, reaching peak responses (9325.0 ± 1178.8 nA) at 10^−5^ M (Figure 4A). EC50 value for rhynchophorol was calculated as 1.779 × 10^−7^ M (Figure 4B), indicating that RpalOR32 responded with high sensitivity to rhynchophorol.

### 3.5 RpalOR25 Shows no Detectable Responses to the Tested Compounds

**FIGURE 5:**
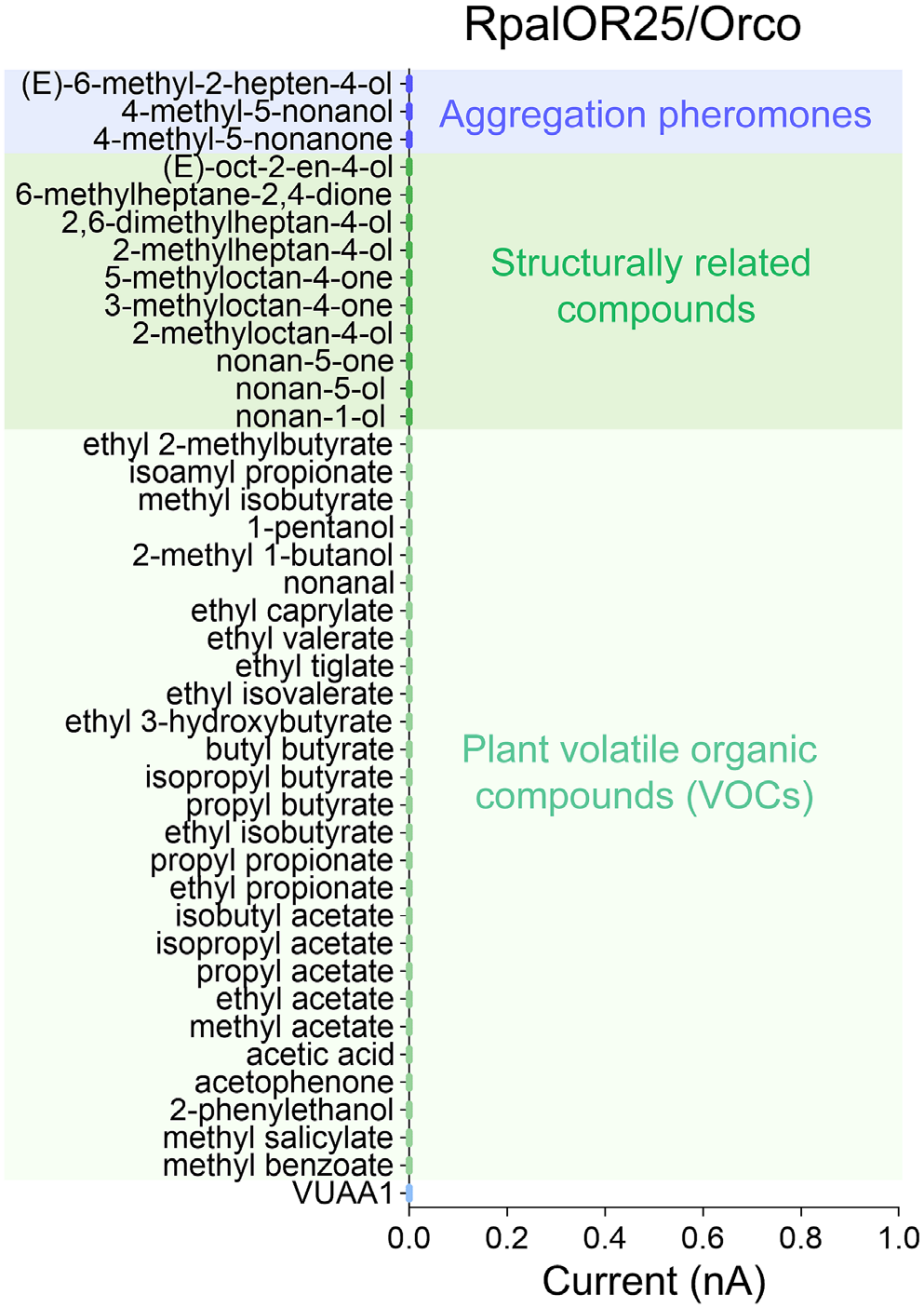
Functional characterization of RpalOR25. RpalOR25/RpalOrco‐injected *Xenopus* oocytes fail to respond to the tested compounds (10^−4^ M) (*n* = 6).

We further assessed RpalOR25's response to the same panel of 40 compounds using the *Xenopus laevis* oocyte heterologous expression system. Oocytes co‐expressing RpalOR25 and RpalOrco failed to respond to any of the tested compounds, including the Orco agonist VUAA1 (Figure 5) (Jones et al. 2011).

### 3.6 Evolutionary Distribution of Functionally Characterized Beetle PRs

**FIGURE 6:**
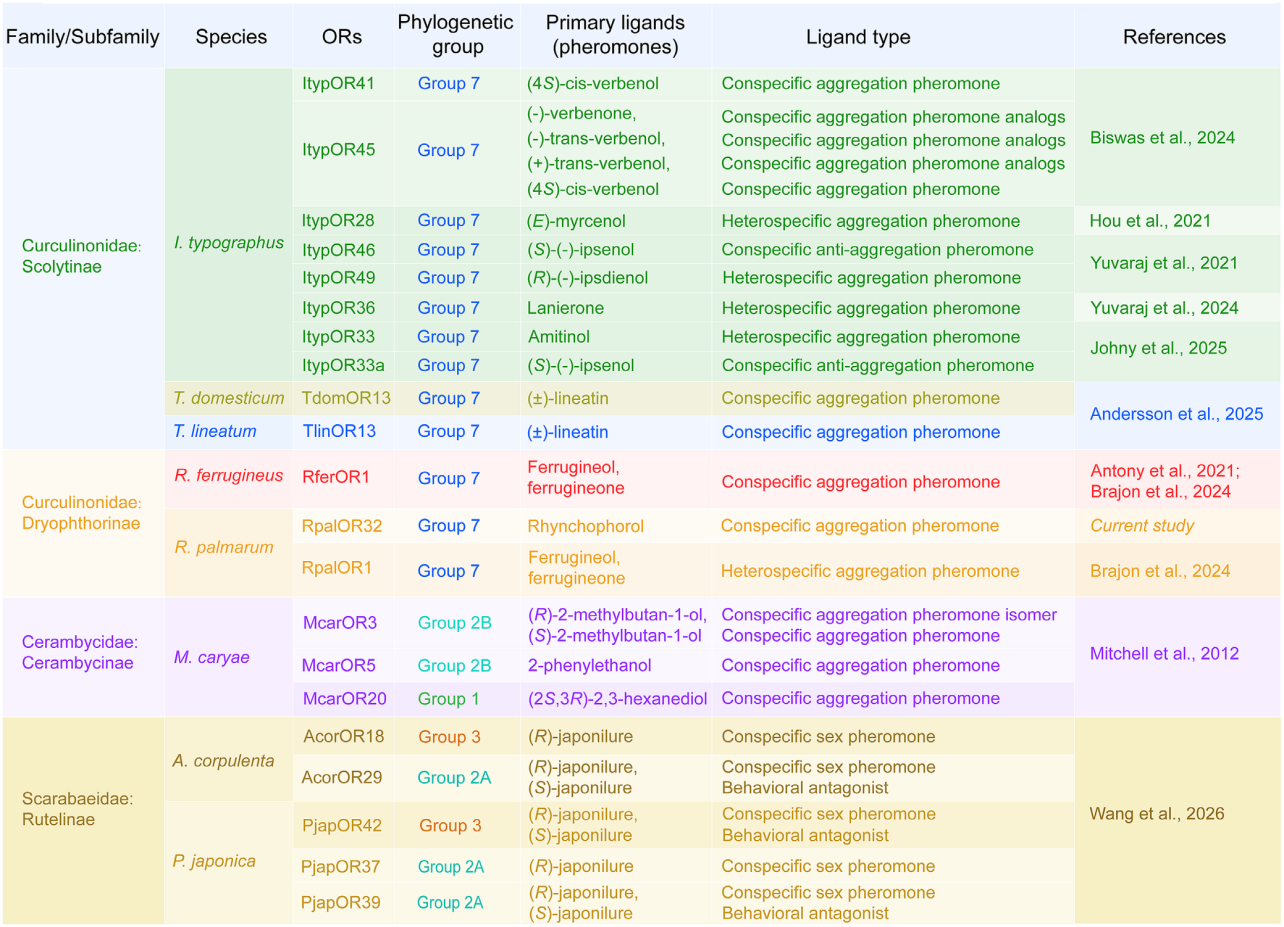
Summary of the characteristics of known beetle PRs. PR names, their primary ligands, their ligand types, and their phylogenetic distribution are summarized here.

To provide a comprehensive overview of coleopteran PRs, all functionally characterized PRs reported were summarized (Figure 6). These PRs were distributed among several lineages within the Curculionidae, including two palm weevils ( *R. ferrugineus* and *R. palmarum* ) from Dryophthorinae and three bark beetles (*I. typographus*, *T. domesticum* , and *T. lineatum* ) from Scolytinae, as well as a cerambycid beetle, *Megacyllene caryae* , and two scarab beetles, *A. corpulenta* and *P. japonica* . Together with RpalOR32 characterized in the present study, and ItypOR33a, a population‐level variant of ItypOR33, 21 PRs have been identified in total in Coleoptera. Among them, 13 curculionid PRs clustered in Group 7 (Figure 1), whereas the three cerambycid PRs were located in Group 1 and Group 2, and the five scarab beetle PRs were located in Groups 2A and 3 (Figure 6). These receptors primarily respond to pheromones and related compounds, including con‐ and heterospecific (anti‐) aggregation pheromones and their structurally related compounds.

## 4 Discussion

We identified the first *R. palmarum* OR tuned to the male aggregation pheromone rhynchophorol. This pheromone elicited an exceptionally strong response from RpalOR32 when expressed in *Xenopus* oocytes. RpalOR32 is the most highly expressed OR in the antennae of both male and female *R. palmarum* (Gonzalez et al. 2021), and it belongs to Group 7 of coleopteran ORs, which contains all the PRs characterized to date in Curculionidae. The expression pattern, phylogenetic position, and high sensitivity and selectivity to rhynchophorol collectively suggest that RpalOR32 plays a pivotal role in mediating aggregation pheromone signalling in *R. palmarum* .

Our study confirms that OR genes with high expression levels in antennae are ecologically relevant to insects. Evidence from multiple weevil and bark beetle species shows that targeting the most highly expressed receptors is effective for identifying PRs in beetles. Such a pattern has been observed in *T. lineatum* , *T. domesticum*, and *I. typographus*, where highly expressed ORs were confirmed as PRs (Andersson et al. 2025; Biswas, Sims, et al. 2024; Johny et al. 2025). Similarly, in *R. palmarum* , the third most highly expressed OR, RpalOR1, responded to the heterospecific aggregation pheromone compounds ferrugineol and ferrugineone (Brajon et al. 2024). However, the second highly expressed OR in *R. palmarum* antennae, RpalOR25, did not respond to the pheromone compounds tested in this study, despite its high expression level and localization in Group 7, with it being close to RpalOR32. By comparing the predicted binding pockets of these two ORs, we identified non‐conserved amino acids, supporting a possible functional divergence. However, we cannot completely rule out that RpalOR25 is a PR, as the RpalOR25‐RpalOrco injected *Xenopus* oocytes did not show any response to VUAA1—an Orco agonist usually used as a control that should induce an electrical response (Jones et al. 2011). It is possible that RpalOR25 expression was not successful in the oocytes or that it may not form a functional Orco/OR heteromer. Alternatively, RpalOR25 proper functioning may require co‐factors such as odorant‐binding proteins (Antony et al. 2018), sensory neuron membrane proteins (Johny et al. 2018), or other accessory proteins that are not present in the *Xenopus* oocytes. Anyhow, given the high antennal expression of RpalOR25, it is suspected to play an important ecological role in *R. palmarum* . Subsequent work could use other expression systems, such as the *Drosophila* empty neuron system that offers a neuronal background (Kurtovic et al. 2007), and could integrate ecologically significant compounds for *R. palmarum* as potential RpalOR25 ligands.

Although RpalOR32 fell within Group 7 of coleopteran ORs, its phylogenetic position was separate from those of previously described Curculionidae PRs, except for the recently characterized ItypOR33 (Johny et al. 2025). This further supports the hypothesis that the ability to detect pheromones evolved multiple times within the Curculionidae, a finding consistent with previous observations (Andersson et al. 2025; Antony et al. 2021; Biswas, Sims, et al. 2024). It is also interesting to notice that RpalOR32 and ItypOR33, although they both cluster within the same subclade in Group 7 and may have a recent common evolutionary origin (Figure 1), recognize structurally unrelated ligands (rhynchophorol versus amitinol, Johny et al. 2025). In accordance with their divergent function, their amino acid sequence conservation is quite low, with only 36% identities, and they present only 7 conserved residues out of 25 and 17 (respectively for RpalOR32 and ItypOR33) within their predicted binding pockets (Appendix S5). Further docking studies coupled to directed mutagenesis may help understand the structural bases of their functional divergence.

Clearly, the Curculionidae PR system differs from moths (Lepidoptera) which use sex pheromones, where PRs cluster together in two clades (Bastin‐Heline et al. 2019; Liu et al. 2013; Zhang et al. 2024). Our results not only suggest that PRs evolved multiple times within Curculionidae, but also within the same genus. Whereas *R. palmarum* and *R. ferrugineus* both belong to the subfamily Dryophthoriae and are from the same genus *Rhynchophorus*, their receptors tuned to their respective conspecific aggregation pheromones (RpalOR32 and RferOR1) appear to be distantly related. Yet, only one study has been conducted at the genus level in Coleoptera, revealing PR ecological conservation: the ortholog PRs TdomOR13 and TlinOR13 from *Trypodendron* spp. ambrosia beetles have been shown to be both involved in sensing their conspecific pheromone lineatine (Andersson et al. 2025). In this particular case, these two species use the same compound as their aggregation pheromone (i.e., lineatine). In *R. ferrugineus* and *R. palmarum* , the aggregation pheromones are different. One has thus to distinguish between PR tuning (ligands) and PR ecological function (conspecific pheromone detection) for evolutionary considerations. Orthologous PRs in the two *Rhynchophorus* species have been shown to exhibit strong conservation of response spectra: RferOR1 and its ortholog RpalOR1 share highly similar predicted tertiary structures and are both tuned to the *R. ferrugineus* aggregation pheromone components and chemically related compounds (Antony et al. 2021; Brajon et al. 2024). Such conserved ligand tuning, coupled with divergent ecological roles (detection of the conspecific pheromone versus detection of a heterospecific pheromone), suggests that certain OR functions are maintained under stabilizing selection, preserving ancestral recognition mechanisms critical for interspecific communication. Collectively, these findings highlight a dual evolutionary pattern in palm weevil aggregation pheromone communication: retention of ancestral receptor functions that ensure recognition of conserved chemical cues critical for intra‐ and interspecific signalling, alongside species‐specific recruitment and diversification that facilitate adaptation to changing pheromone signals and ecological contexts. This dynamic balance between functional conservation and ecological diversification indicates the complex evolutionary architecture of the palm weevil PR systems. Future functional analyses of PRs and their orthologs, such as the RpalOR32 ortholog in *R. ferrugineus* , will be crucial for validating the proposed evolutionary framework and deciphering the molecular basis of pheromone recognition diversity in palm weevils.

Although RpalOR32 exhibited detectable responses to several structurally related compounds of the aggregation pheromone, its response to rhynchophorol was markedly stronger than to any other tested compounds, indicating a high degree of ligand selectivity. Similar highly specific aggregation PRs have been identified in *I. typographus*, *T. lineatum* , and *T. domesticum* (Andersson et al. 2025; Biswas, Sims, et al. 2024), but RferOR1 from *R. ferrugineus* did not exhibit such specificity, responding to compounds structurally related to pheromones and, surprisingly, to several plant VOCs, a profile also observed for RpalOR1 (Antony et al. 2021; Brajon et al. 2024). In our study, RpalOR32 did not respond to any of the 27 plant VOCs tested. Different heterologous expression systems may partly influence the observed receptor tuning profiles across studies (*Xenopus* oocytes in this study, *Drosophila* neurons in RferOR1 and RpalOR1 studies). Although *Xenopus* oocytes and *Drosophila* neurons typically identify the same primary ligands (Guo et al. 2022; Wang et al. 2018; Zhang et al. 2023), secondary responses may vary due to differences in receptor microenvironment, accessory protein interactions, receptor folding dynamics, or ion channel efficiency. Regardless, the high specificity of RpalOR32 we observed here matched with the responses of the most abundant OSN type characterized on *R. palmarum* antennae by electrophysiology (Saïd et al. 2003). According to this in vivo electrophysiological analysis, two other *R. palmarum* OSN types also respond to the aggregation pheromone rhynchophorol. One type displays a high specificity but a low sensitivity to rhynchophorol, while the other type responds to both rhynchophorol and ferrugineol, with a high response threshold (Saïd et al. 2003). Considering that OSNs usually express only one OR type, these findings suggest that, in *R. palmarum* , at least two additional ORs may contribute to the detection of rhynchophorol. However, only a limited number of PRs have been functionally characterized in *R. ferrugineus* and *R. palmarum* , and it remains unclear whether each species possessed both narrowly (as RpalOR32) and broadly tuned (as RferOR1) aggregation PRs. Future studies involving deorphanization of additional PRs will be essential to establish receptor‐sensillum‐neuron mapping at the peripheral level and to achieve a comprehensive understanding of aggregation pheromone recognition in these two species of palm weevils.

*Rhynchophorus palmarum* is the most destructive palm weevil species in the Americas because direct feeding damage by larvae kills palms of agricultural and ornamental importance. Additionally, this species vectors a palm‐killing nematode, the red ring nematode, that causes a lethal and incurable palm disease known as red ring disease. *Rhynchophorus palmarum* has expanded its range within the Americas, and invaded California (USA) sometime around 2014 (Hoddle et al. 2024). Use of traps loaded with aggregation pheromone and fermenting bait has been widely used for monitoring the spread of this palm pest, in California (Hoddle and Hoddle 2017; Hoddle et al. 2025). Although the aggregation pheromone plays a fundamental role in *R. palmarum* sexual communication and attraction to feeding and oviposition sites, as well as in pest control, there was no information on the molecular mechanisms of its recognition in *R. palmarum* . Our findings thus provide a significant advance in understanding the molecular basis of pheromone communication in *R. palmarum* , pheromone divergence and speciation in *Rhynchophorus*, and the evolution of PR in Curculionidae. Results presented here also pave the way for the development of PR‐guided strategies for sustainable *R. palmarum* control, either targeting RpalOR32 for the design of new attractive semiochemicals in a so‐called reverse chemical ecology approach, or providing a new receptor interface in OR‐based biosensors able to early monitor pest infestation by the detection of the weevil pheromone in the fields, as recently developed using RferOR1 from *R. ferrugineus* (Hoddle et al. 2024; Mimura et al. 2026).

## Author Contributions

E.J.‐J., B.A., and N.M. designed the research. M.H. collected weevil samples. A.P. performed sequencing. B.A. created the *R. palmarum* antenna transcriptome. S.Z. performed the experiments and analysed the data. E.J.‐J. and S.Z. wrote the paper. E.J.‐J., G.W., B.A., and N.M. reviewed and edited the manuscript. All the authors approved the final version of the manuscript.

## Funding

This study was supported by the CAAS‐INRAE‐AgroParisTech Associated International Laboratory in Plant Protection BIPi and received funding from Sorbonne Université, INRAE, the French program ‘Cultiver et Protéger Autrement’ (grant ANR‐20‐PCPA‐0007), and the French National Research Agency (grant ANR‐25‐CE20‐2183).

## Ethics Statement

The authors have nothing to report.

## Conflicts of Interest

The authors declare no conflicts of interest.

## Supporting information


**Appendix S1:** Phylogenetic tree of Coleoptera ORs.
**Appendix S2:** Nucleotide sequences of *Rhynchophorus palmarum* ORs and Orco.
**Appendix S3:** Chemical compounds used in the study.
**Appendix S4:** Amino acid sequences of *Rhynchophorus palmarum* ORs identified in the newly assembled transcriptome.
**Appendix S5:** Comparison of RpalOR32 and ItypOR33 sequences and 3D structures.

## Data Availability

The list of compounds used in this study is provided in the Appendix S3. The RNA‐seq data generated in this study have been deposited in NCBI (BioProject PRJNA656150, SRA accession numbers: SRR29255395 and SRR29255396 for male and female antennae, respectively). RpalOR expression data are from Gonzalez et al. (2021) and are available at: https://www.nature.com/articles/s41598‐021‐87348‐y. The nucleotide sequences of RpalOR32, RpalOR25, and RpalOrco are available in the Appendix S2, and all OR sequences identified in the new transcriptome are provided in Appendix S4.
