# Supplementary material for: A Highly Expressed Odorant Receptor Detects the Aggregation Pheromone Rhynchophorol in the Invasive American Palm Weevil
Source: Mol Ecol. 2026 Aug 9;35(15):e70504. doi: 10.1111/mec.70504 (PMC13454776; doi:10.1111/mec.70504)
Supplement: Supplementary file 1 [file MEC-35-e70504-s001.docx]

**Supplemental Information for:**

**A highly expressed odorant receptor detects the aggregation pheromone rhynchophorol in the invasive American palm weevil**

Sai Zhang, Nicolas Montagné, Binu Antony, Guirong Wang, Mark Hoddle, Arnab Pain, Emmanuelle Jacquin-Joly

**Appendix S1. Phylogenetic tree of Coleoptera ORs**

**Appendix S2. Nucleotide sequences of *Rhynchophorus palmarum* ORs and Orco optimized for *Xenopus leavis* expression (optimization conducted by Synbio Technologies)**

>RpalOR25

ATGGGGATGTTCAACTTCCTGAAGGTGAACTTGTTCGGCCACTGTATGGAGCTGTACTACATCATGG

GGATGATGCCTAACTTGCCTGACGGATCAAACAAAGCCGGCTGGATATTCTCCACATACTCCTTCCTGCTGTA

CTACAGCAGCTGCGTTTGCAACTCCTTCGAGTTCGTGAACCTGGTGCAGATCTTCATGGACAAGAAGGAGAGA

AACCTGGAGGCCGCTATAACCAACTACATGCTGGCTTCATTACACGCCGCCGGATTACTGAAGTCAGGACTGT

CCAAGTCCAAGATCGGAGCTAAGTACTTCCAGAACGTGGTGAACTTCGAGAGAAAGGTGTACGAGTCCGGAAA

CAAGGAGGTGATCGACGTGTACGAGAACAATTGTAAGGCCTTCAGAGCTACAGCCGCTAGATACTTCACCTCC

GTGTTCCTGGTGATCGTTTGTTACGGGTCCTCCTCACTGATCAGATCAAAGGAGCTGGTGGTGATCGGGAACT

TAACATACGAGACCAGACAGGTGCCTCTGAAGATTTGGTCCCCTTTCAACCAGTACACCCAGTACTTCTGGGC

TTACCTTTGGGCTATCTCCATCGGGACAATACTGGCTTTCTACTTCGTTGCCGGGGATCTTATCTTCTTCGGG

TTCACCATCTTCGCCCTTTGTAGACTGGAGATCCTGAAGTACTTCATCATGAACTTCTACAAGAAGGCCAACG

AGATCAGATACCACCTGAATTGCGACACCGTGGAGTCATACAGAATCCTGCAGAGAGAGTGCATCATCATGCA

CCAGGAGATCATCTCCTACATCGAGGATCTGAACTCCCACATCAAGCACATGATGCTGATGGACTTCCTGCCT

GGATCAATACAGTTAGCCGCTCTGCTGTACCAGCTGATCAACAACCTGAACGTGATCCAGATGGTGCTGATCG

GAGAGTTCCTTCTGACACTGACCGCCAGATTCTTCATCTATTGCCTGAACGCCAACAAGATCAACGAGCAGTC

ACAGATGATCGGGACAGTTTGGTACCAGATCGATTGGACCATCTTGCCTGAGGACGTGAAGAGAAACATCGTG

ATCTGCATCAACAGAACCCAGAAGCCTCTGGCTATCTCAGTGGGAGATTTCCAGGATATCAGCCTGGTGACAT

TCGTGTCAATCATGAAGGGGGCCTACACCTACATGATGTTCCTGACCACCTTCTAA

>RpalOR32

ATGGCCATCTTCGACCTGAAGGTTGATCTGTTCGGCAGATGCGTGGAGTTATACTACCTGATGGGGA

TCATACCTCTGCTGCCTGATAAGAGAAACAAGCAGGGACTGGGGTACAGAATCTACTCATTCGCCATGCTGTA

CTCAGGGTACATTTGCACAGGGCTGGAGTTCGTTAACCTGGTGCAGTCAATCCTGTCCAGAAACACATCCCTG

GAGATCATCATCACCAACTACATGCTGGCTTCACTTCACACAGCCGGATTACTGAAGTCAAGACTGGGAAACT

CAAAGAAGGGAGCCGAGTATCTGCAGAAGATCATCGACTTCGAGAAGAAGGTGTACGCTTCCAGATCCAAGGA

CCTGATCAACCTGTACAAGAGAAACGGAGCCGCTTTCAAGATGACAGCCGCCGGATATTTCACCGGAGTTTTC

ATGGTGGTGGTTTGCTACGGATCCTCATCACTGTTCAGATCACAGGAGTTCGTGCTGATCGGAAACGTGTCAT

ACGAGATCAAGCAGCTGCCTATCCCTATTTGGTCCCCTTTCAACCAGTACACCCAGTACCTGCTGACATACCT

TTGGTCACTGGTGATCGCCACAGAGTTAGCTTTCTACTTCGTGGCCGGAGATCTGACTTGTTTCGGGTACACA

ATCTTCGCCCTTTGCAGACTGGAGATGCTGAAGTACTTCATCATCGAGTTCTTCACCAAGGCCAAGGAGATCC

AGCAGAACCTTAAGTGCGACGAGATCGAGTCCTACAGAATCCTGCAGAGAGAGTGCATCATCATGCACCAGGA

CATCATCTCCTACATCGACGACCTGAACTCCCAGATCAAGTACCTGATGCTGATGGACGTGTTACCTGGATCA

ATCCAGATAGCCGCTCTGTTATACCAGCTGATGAACAACCTGAACGTGGTGCAGATGGTGCTGATAGGAGAGT

TCCTGCTGACACTGACAGCCAGATTCTTCATCTATTGCCTGAACGCCAACAAGATCAACGAGGAGTCACAGCT

GATCGGAACCGTTTGGTACCAGATCGATTGGACAGTGTTGCCTGAGGACGTGAAGAGAAACATCGTGCTTTGC

ATCAAGAGAACCCAGAAGCCTCTGTCCATCACAGTTGGAGATTTCCAGGAGATCAGCCTGGTGACATTCGTGT

CAATCGTGAAGGGAGCCTACACCTACATGATGTTCCTGACCACCTTCTAA

>RpalOrco

ATGATGAACACCTTCAAGGTGACAGGACTGGTTGCCGACCTTATGCCTAACATCAGACTGATCCAGG

CTTCAGGACACTTCATGCTGAACTACCACGCCGATAACTCAGGAGCTTTACACGGACTGAGACTGGGATATTG

TTGCATGCACCTGCTGTTCGTTCTGGTTCAATTCGGCTGTATCTTCGGGAACCTGGTGAAGGAGAAGGACAAC

GTTAACGATCTGGCCGCTAACACCATCACAATCCTGTTCTTCACCCACTGCCTGACAAAGTTCGTGTACTTCG

CCGTGAGATCCAAGCTGTTCTACAGAACCCTGGGGATTTGGAACCAGGCTAACTCACACCCTATCTTCATCGA

GTCAAACAACAGATACCACGCTCTGGCTCTGAAGAAGATGAGAAACCTGCTGTACATCATCATGATCGGGACC

ATCTTCTCAGCTTCAGCTTGGACAGGGATCACATTCATGGGAGAGTCAGTGCACTACATCAAGGACCCTAACA

ACGAGAACGAGACCATCTCAGAGGAGATCCCTAGACTGCTGATCAAGTCTTGGTACCCTTTCAACGCTATGTC

AGGGATGCCTTACTACATCGCTCTGGTGTTCCAGGTGTACTACGTGCTGTTCTCACTTCTGCACGCTAACCTG

CTGGATTCACTGTTTTGCTCTTGGCTGATCTTCGCTTGCGAGCAACTGCAACACCTGAAGGAGATCATGAAGC

CTCTGATGGAGCTGTCAGCTTCACTTGATACCTACGTGCCTAAATCAGCCGATCTGTTCAAGGCTCCTAACTC

AGCTCACTCACAGGACAACCTGATCGAGAACGAGTACAACTCCAAGAACGACGAGCTGAACCTGAAGGGAGTT

TACTCCACAAGACAGGAGCTGGGAAATCTGACCTTCAGATCAGGAGCTCTGCAGACATTCGGACAAGGAGGAG

GAGGAGTTGGACCTAACGGACTGACAAAGAAGCAGGAGCTTATGGTGAGATCCGCCATCAAGTATTGGGTGGA

GAGACACAAGCACGTGGTTAGACTGGTTACAGCTATCGGAGACGCTTACGGAGTTGCTTTACTGCTGCACATG

CTGACAGCTACAATCATGCTGACACTGCTGGCTTACGAGGCTACAAAGATCGACGGAGTTAACGTTTACGCCG

CTACAACACTGGGGTATTTACTGTACTCACTGGCTCAGGTGTTCCACTTTTGCATCTTCGGGAACAGACTGAT

CGAGGAGTCATCATCAGTGATGGAGGCCGCTTATTCTTGTCACTGGTACGACGGATCAGAGGAGGCTAAGACA

TTCGTGCAGATCGTTTGCCAGCAGTGTCAGAAAGCTCTGTCAATCTCAGGGGCTAAGTTCTTCACCATCTCCC

TGGATCTGTTCGCTTCAGTTCTTGGAGCTGTGGTGACATACTTCATGGTGCTGGTTCAGCTGAAGTAA

**Appendix S3. Chemical compounds used in the study.**

|  | Chemicals | CAS number | Purity | Manufacturers |
| --- | --- | --- | --- | --- |
| Aggregation pheromones | (E)-6-methyl-2-hepten-4-ol (Rhynchophorol) (*R. palmarum* aggregation pheromone) | 4798-62-3 | >98% | ChemTica Int., Costa Rica |
|  | 4-methyl-5-nonanol (Ferrugineol) (*R. ferrugineus*) | 154170-44-2 | >98% | ChemTica Int., Costa Rica |
|  | 4-methyl-5-nonanone (Ferruginrone) (*R. ferrugineus*) | 35900-26-6 | >98% | ChemTica Int., Costa Rica |
| Aggregation pheromone analogs | nonan-1-ol | 143-08-8 | >98% | Sigma-Aldrich |
|  | nonan-5-ol | 623-93-8 | >99.5% | Fluka |
|  | nonan-5-one | 502-56-7 | >98% | Sigma-Aldrich |
|  | 2-methyloctan-4-ol | 40575-41-5 | >95% | Synthetized |
|  | 3-methyloctan-4-one | 20754-04-5 | >95% | Synthetized |
|  | 5-methyloctan-4-one | 6175-51-5 | >95% | Synthetized |
|  | 2-methylheptan-4-ol | 21570-35-4 | >95% | Synthetized |
|  | 2,6-dimethylheptan-4-ol | 108-82-7 | >95% | Synthetized |
|  | 6-methylheptane-2,4-dione | 3002-23-1 | >98% | Sigma-Aldrich |
|  | (*E*)-oct-2-en-4-ol | 4798-61-2 | >95% | Synthetized |
| Plant volatile organic compounds (VOCs) | methyl benzoate | 93-58-3 | >99% | Sigma-Aldrich |
|  | methyl salicylate | 119-36-8 | >99% | Sigma-Aldrich |
|  | 2-phenylethanol | 60-12-8 | >99% | Sigma-Aldrich |
|  | acetophenone | 98-86-2 | >99% | Sigma-Aldrich |
|  | acetic acid | 64-19-7 | >99% | Prolabo |
|  | methyl acetate | 79-20-9 | >99% | Sigma-Aldrich |
|  | ethyl acetate | 141-78-6 | >99% | Sigma-Aldrich |
|  | propyl acetate | 109-60-4 | >99% | Sigma-Aldrich |
|  | isopropyl acetate | 108-21-4 | >98% | Sigma-Aldrich |
|  | isobutyl acetate | 110-19-0 | >99% | Sigma-Aldrich |
|  | ethyl propionate | 105-37-3 | >99% | Sigma-Aldrich |
|  | propyl propionate | 106-36-5 | >99% | Merck |
|  | ethyl isobutyrate | 97-62-1 | >98% | Merck |
|  | propyl butyrate | 105-66-8 | >95% | Sigma-Aldrich |
|  | isopropyl butyrate | 638-11-9 | >99% | Sigma-Aldrich |
|  | butyl butyrate | 109-21-7 | >98% | Sigma-Aldrich |
|  | ethyl 3-hydroxybutyrate | 5405-41-4 | >99% | Sigma-Aldrich |
|  | ethyl isovalerate | 108-64-5 | >98% | Sigma-Aldrich |
|  | ethyl tiglate | 5837-78-5 | >98% | Lancaster |
|  | ethyl valerate | 539-82-2 | >99% | Sigma-Aldrich |
|  | ethyl caprylate | 106-32-1 | >98% | Fluka |
|  | nonanal | 124-19-6 | >95% | Sigma-Aldrich |
|  | 2-methyl 1-butanol | 137-32-6 | >98% | Fluka |
|  | 1-pentanol | 71-41-0 | >99% | Sigma-Aldrich |
|  | methyl isobutyrate | 547-63-7 | >99% | Fluka |
|  | isoamyl propionate | 105-68-0 | >98% | Sigma-Aldrich |
|  | ethyl 2-methylbutyrate | 7452-79-1 | >99% | Acros |

**Appendix S4. Amino acid sequences of *Rhynchophorus palmarum* ORs identified in the newly assembled transcriptome**

>RpalOR48

MVKVVDDTLLLKTIKIYLIICGLWQRSFQQNRLNILYRIYGEILKYIVLLLVVSFLTEIS

IEIFNFDTSQKPDRLIKNTAFVISYCESIVIIIIYKKNNVLEMLDMIQEKEKVVMESKEV

ELIKIYKKEVRYCGILNLILFSICGGTGIILDLGTVLRYTESTENTNIQSMLDFGLELWL

PIDKTEHPLLVTVINLVAAPIGAATFAIIQMILLTMMTFPSVHLQILCFRLKQMKADVGQ

YFLRQKLKHYIIEQKFLITYTKTLNSAVRYVILMQFILVSLNVAAVAVNATKTENLTETI

FWILYFIVLVIQTFSLSCTANNINEQNYLIADALYQSEWYDFDNKIKDMLKLMMIQAQQP

LVLTIGPFNPLTTQTGLKIMNIAYSYVTLMTNVQ

>RpalOR45

MKFPENKLLKISMYLFTMVGVWPIIQWESEKSKRIYNITSAILYYYYLEYIFRAYFKLYL

LLTTDTLYIEEILGNLCITLIYTVSMLRLKAFNTQAIRTLFINVIDEEKSILKNPDPEIR

KMYMSSVRDTVKYNILFLFNGWVVSFLYFIHPFFMELPTEVVNNQTVTLRTLPLSTWWPI

DIQEHYWLAYCWNVFDGTLGSSYVINSDILTFSLIVFALSQVKILSYKLRQFPTYRKTGN

EEEDNKNTLLEFVDLVKEHKKIISYIDTFNEGMKYVMLFDFLQCSVQLATITLQLLVMEI

NVQNVIFVGEFLLTMLIRLVIYYFNANEVIYQSRHLALDVWNCRWYEENRKLKRIMLIFI

IRSQKPLRFLIGPFGVMSLETFISILKATYSYVMIFINTNT

>RpalOR18

QLIISAIGVLITCCLIATKTYICVRRGLIDVLDEVVEYEEKLMRSDNEDVKNMYFTKALF

CNNLSRGQALIQIFCALAMYYLGIVGDQELIAYNKANNATIDSYYWYQFWFPGFKLDHLF

WIYTVNLMFGWSACSANVVCHSIISTLMIFATVQLQILNIRLRGFVEEGDDIKSKTAELK

GYVKEHQTLISYVHKLNMEIRFIVLLQFILNSIEIATCVTGLLKDIPTTHVIFLLSYICL

TSYQLAMTSWNANEILEQ

>RpalOR68-like

MTGKFIKSLKIMMIVSGYWEKPLTGSIIVNKLYYAYYILSQTILVTFCIELFLGLLDHII

KGHDKGIIIASAGVVITCLVIVLKAYICFKRGLIKILDEIVKYEELISKTDDAEIKQAYW

SKVQFTNVMCITQALAQCWASGSLFYLGITGDQTIIQYNKIHNTTVDSPYWYQIWFPGTK

LDHRVTFYTLNVIFCIFASLSNVTAHSIIATLMIFAATQLEILNIKLKKFIQDKNDKVDD

KIPELKEYVKQHQDLIKYVCVLNKKINIIILMHFLLNSVEIASCVTGLLKNVLTSQRVFL

MSYINIVTYQLFVTSWNANEVLEQSTAISDAIYTSNWHLLDKDGKVICQIMMQRAQRPLK

LTIGPFGPMTNRTGLMVIKAAYSYFSLMNGGYSQN

>RpalOR5

MIIKRDYVEKIITNYMLASLHLAGIIKSLMTKSKKGGWLLCRMIEFEQKVYKSNDKQILA

LYKRNIRAFRSLSGWYFVGIVVVVACYASSSIFRPKEYMIHGNETVEVKQVPMMIISPFD

QHDQHMSAFFWSTCTGGFLAIYFVTGDLMCFGFTIFAFCQIDILKYFIGNFSKKSREIEN

YHRCEAGHAFRIVQRECIIMHQDIIRYVEELNASMKYLMLVDFLPGSIQIASLLYQLMNN

LNAIQIVLLGIFIITLIIRLHIYCNNANKITVESEMIGDMWYQSDWTEMPLDVQRNMMIC

IARTQKPLTITVADFQNASLATFVAIMKATYTYMMFLTTF

>RpalOR33

RMVDASESVFSALLLLQYIVSIISICNGVFQLTHTDSSATLSVVCNSLFVFNVLFEIAVC

WFAGLFTAKSVELSTAYYMYDWINSSESVKKSLLILTHRSQQPIYMTAGTVVRLSMASFL

QVIKAAYSYFTLMQNLYNKSI

>RpalOR65

MSLPFIMLYHPKKHEAFYDTLNFLKMLFFYPKEQRGIVVTQNLQWFLARFLTAFNFFFIL

LHTVVCLKERRFGGLSDDVIYGAGFACTLLSVKLVETKHKKWSDFMRRLTDFTIYGKPNE

YDQVVKKLNLLSKMVNRYVYFSVIVFYCLQLWNRETCERNNMEKGIDDMCGVILPFWWPY

PLTPVKTIIISTIQTIAALIYVPVMAIVIVLPTEIVEIIRLRVKHFCDLLTNTFDESSTE

SEENGRLRHCIRYHVELLEIGMEASENISLLMAHVVFTASVEVASTVYSYIMTPYQYVYL

FITLAWLMGTGLACRSGQLLQDTYSDILEAAYLSKWYHASISNKKILLFLMMRTSKTIPV

KALPCGVFSYGVFVNIIKGTYSFLTLMVGK

>RpalOR60

SSTLIDYVFWTSFTVLVLIQLSIMTWHANEIQLQSLSIADSVYESNWYYQKISIKRIIMV

VMMRAQKPIYIHVGLLFPMTLACALTTIKAAYSYITLILQFDSQ

>RpalOR41

FLDNSVDLYGMTYLCQLFASTTTFCTGLFLLTVTEMSFEYFYLLSYVVAQSFLLLTPCWS

ATEMTRKSEKIPMAAYSTDWIHGTTYFKRMLMLIIIRSQKPMKFYAGNFFELSLETYVSI

SRASYSYFALLSKMTNEK

>RpalOrco

MMNTFKVTGLVADLMPNIRLIQASGHFMLNYHADNSGALHGLRLGYCCMHLLFVLVQFGC

IFGNLVKEKDNVNDLAANTITILFFTHCLTKFVYFAVRSKLFYRTLGIWNQANSHPIFIE

SNNRYHALALKKMRNLLYIIMIGTIFSASAWTGITFMGESVHYIKDPNNENETISEEIPR

LLIKSWYPFNAMSGMPYYIALVFQVYYVLFSLLHANLLDSLFCSWLIFACEQLQHLKEIM

KPLMELSASLDTYVPKSADLFKAPNSAHSQDNLIENEYNSKNDELNLKGVYSTRQELGNL

TFRSGALQTFGQGGGGVGPNGLTKKQELMVRSAIKYWVERHKHVVRLVTAIGDAYGVALL

LHMLTATIMLTLLAYEATKIDGVNVYAATTLGYLLYSLAQVFHFCIFGNRLIEESSSVME

AAYSCHWYDGSEEAKTFVQIVCQQCQKALSISGAKFFTISLDLFASVLGAVVTYFMVLVQ

LK

>RpalOR7

MIIFWIRNTTSIQTLAAIGYVIFICLMGQLKSILLFKHRREFKKLIQDLEQKYFQPKNIS

EEQLVKDTFSFLWKIKTALITISYISVTSLLILPLLYDKERSLPIQAWYPFDKFLTPNYQ

IVYIHQAIAIYYISSINIYVDVILAGLSTFIGLQCDLLCERIVNLGTFGMEKQIKRKFVD

CIDHHYTIIEFLKTTENVFGQIYFGQIFASTLALCMDLFLLSLTEPKTFQFFYLVVYQLA

ISNLLFVPCWFVSEMTKKSENIPNAAYNCNWTHLSISFKKELMIFIRRSQRPLKFYAANF

FEISVQTFLTF

>RpalOR47

MYEPKKGEMFYSTLTSLRWFYLYPSKKDSKDWKLYSIKAVIVRIITIFGFIECWLHLIMS

IIEDAPVNLAEDIEGIVGYGNSVFVCCTFQYMCIHWSKFFKDITDHTRYGKPPDYDKTVQ

KTNMFSSLYYVFTTVSVILYGIVSFLDTKQCKLMNELNGTHEICDAVTPLWWPSAYMNPW

VKAFLTFYLLLSCEYYLPASGVVSVIVWESTILIEAKINHLKCLFRECLNDDNPKKRRQK

FNFCIQYHHEIYRLCDEINRLSKWIIGQLSFAAAVVIGCIANQMIKQYSIGSMFHLVGYM

IVVFLACHTGQVIKNKTYDVQDALYESKWVDADREMIRDVQFVILRCQKPAVLKAIPLGT

FDYSVWIVIIKTSYSYVTLLTKQDN

>RpalOR26

MGIVDKKVVNMAEDIEGITGIGYATFVCSIFQYNFNDWCQFFHDVVDTTKFGKPHKFDDV

VYKTNFLSKSCFWLTSLFVIIYSVLSLLDTEQCQSLNQKNGTHEICGTVTILWWPTEHID

SRIKVFISVYQLLAFELFIPTSAIIAFIVLESVMVIQEKSHHLKQLFKESFECNNPVKTC

LRFKFCIRYHNEIFRLCDKLNKLTKWVIGQLAVTAAIVLGCISNRMITEQYYESVVYLCG

YLMIVFVACHAGQILKDETMDIQDALYKADWTKTNKETIRDVQYVMLRCQKPAILKAIPV

GTFDYGVLITIIKTSYSYLTLLNNQV

>RpalOR16

MYQPQKGDMFYSSLTILRWMYLYPSEECSKNWTLYVVKAVIIRVITSFTFLECCLHFIMS

SIDNIPVNLAEDIEGITSLGDSVFVCCLFQYMAKDWSIFFKEVTDIKQFGKPPMFDKAVR

ITNLSSTVYIIGTSVAVMFYGIVSYLNTEQCKIQNDLNGTHEFCDIVMPLWWPSENINPW

VRLVINICLVLSCELYLPTSGIVSFIVWESTILIQAKIAHLKQLFKECLDDNDPRKKRQK

LNICVQYHLEILRLCDELNRLFKWIIGQLSFAAALITGCIVNQMIKQYSIGSMLHLCGYL

LTVFLACETGQIMKDETYDIQDAVYESRWVKSNRDIVGDLQLVMLRCQTPKHLRAIPLGS

FDYSLWIVILKSSYSYITLLTN

>RpalOR9

YGVAIRTIYIAFWLALIAELFRLILQNYDSSIIMSSVGVVVNATKMLVKLLIFSKNGLGN

LFEYVIKKEDETWACNDEDIKRLYRKKVSICRKFMIIQIAGSFGAVIALNIMGIYGNNKL

IQDNRQHNTSTETHLMYQIWFPANKLDHLVTFYTITFLFSWSGIVYSCITHMMFISLLIF

GAVRLQILQIKLRKVANTTQSVDQKKNYSPVKR

>RpalOR12

MIEIIFENRLLYQMNADQRKEMENIINMTQYVMNYYRFAVFVAWVAYNSFPILDSMDDRR

LVIPAYFPWSMANLIVYIVTYVYQSVMLFISASLNTTVDILFAMFCTVVCQEFKILKDHL

QNLNYDQHSSTIKTDIKRNVSFHVELIRATKIIEDTFSYGILSQFAASISVICFTGLELI

L

>RpalOR42

MISGDVKKTDNIITITCCAGVKAIIYSSARIKTIIKRSRSNKNVHKIYWSVKYINKCAIA

IVVIAGGTAGVVATTTIGSNNDKMIAHDKKYYISIIASSIVVATVYYCMSIYCITVKAKK

IYHRCNHHNKGTNIIRKVKHHRIIKVKNDSIKYISSSNIASVMYSATITDIVSISYMVIG

IAWHANIKISVDVSDAAYSSSWYGASGNVKTMIRMVIIRSNKTIGNTTTVISVINAAYSY

VMIMTNSNDI

>RpalOR68

MTGKFIKSLKIMMIVSGYWEKPLTGSIIVNKLYYAYYILSQTILVTFCIELFLGLLDHII

KGHDKGIIIASAGVVITCLVIVLKAYICFKRGLIKILDEIVKYEELISKTDDAEIKQAYW

SKVQFTNVMCITQALAQCWASGSLFYLGITGDQTIIQYNKIHNTTVDSPYWYQIWFPGTK

LDHRVTFYTLNVIFCIFASLSNVTAHSIIATLMIFAATQLEILNIKLKKFIQDKNDKVDD

KIPELKEYVKQHQDLIKYVCVLNKKINIIILMHFLLNSVEIASCVTGLLKNVLTSQRVFL

MSYINIVTYQLFVTSWNANEVLEQSTAISDAIYTSNWHLLDKDGKVICQIMMQRAQRPLK

LTIGPFGPMTNRTGLMVIKAAYSYFSLMNGGNSQN

>RpalOR11

LRLAGQINERTQQNIGQLSFVAAIVMSCIGNQMLKQYSLGALIHLAGYLVAVYLVCQAGQ

AIKDNTESISDSVWFSKWYTDLKLAKDLKIVIQRSQQPVPIRSHLLGDFDYRLLIVIIKT

TYSYLTLLTR

>RpalOR46

MSDNEQLVKLTRLFLIFTGFWIKPLNIRRPWKTLYSVYAALIRILIALIWILLFIELLRL

LIQGYDLEIVVSSVGVLISYTRVEMKFVIFVKNNVGSLYRVVMEKEAEIWGSGDDKIKEL

YVRRVKKCNHFMIALILGTILAVLTLGVTGIIGNNKLIKYNKLYNETLETHLIFQFYFPA

NKLDHLIWYYTIVAVFSWCSFCYNVASHMIYNNLLIFSAIQLELFRVKVTNAIEGSRSAE

ENNRGLKKLIVEHVSIIKFISSLNDKAANVLMLEFFLSSMDIASAIANISELTGTELEWR

LAFITVLLIQILAITWNANEIKEQSLSIAEGMFISDWYLLNKEGRQMTQIIIMRAQVPLA

VTIGPFGPMTTNSALVTLKAAYSYVNLMQ

>RpalOR52

MTDNEQLIKITKWFMILTGFWIKPLDVGLKTKKIYYAYVMCVRIGFVSFWLLLMVELLRL

IIQNYDLEVVMSTLGILVGATKIQVKFIIFIKNKVVDLYEDIIENESKIWRSNNKEIKKL

YVRNVIICRYFMIVQMVGSLFAVTSLNITGILGNKELIKYNHIHNTSLETHFIFQVYFPV

SKLDHLVSYYSLMLLCTWCAFIFNNASHMIITNLLIFCSTRLEVLQIKMRNVVSNRNLEG

QNRRLKKLILEHVSIIQFISTLNHRTKYILMLEFILSSLDVASAAANVTKTKTSDLSWWS

VFFLLLTVQILSLTWNANEIKEQSLSVADNIFKSDWYLLNINSRKSVRLIIMRAQTPLVV

TIGPFSPMTTTSALLMFKAAYSYVSIMQ

>RpalOR37

MDNERLIKLTKWHMIAVGFWKFDNQNKAQSKIIHWLYDVYSKSVPVMYFVVLIGLTIESI

KMCLQRDSLRRISEAVAVLHAVLDAVVKIFIFYNKKIPDLFGYVKKMEADVWNSNDASIK

NHYRQQIRYCRFLCSIVMALVFATGTSFTVAGFVKLSCNDIDINSFMYYLWLPFDKAPYQ

TYLCVLYVILCYNSVIFIVCVKIVLITLIVFVKTHFQILQIRIKKLGSTGNSGELIKSLV

HQHLFLIRYSAKLNDSVKYILLLEYVLNSVNVASIIFQILTIESLIDLLFPVVHSVTITA

EIFALAWTATELKEQSSMVSDAIYESYWYNLDRSSLRLINIMMIRAQSPMVLTIGPFSPM

TTNTAVAIMKAAYSYVMLIMNRYDHE

>RpalOR29

MCIWLQNTKSKLYNAFLLQFIPLRVYGLYPFNGWQRKTYNIYAMIIYMVLVVPVPILALI

YVGKLLNSSNGSINMVEISDSLMVITEMGVNIFKYLDLKLHPRSIKKTMDWLNTPLFNSF

NGKQEHMIERAYNFCRKFYFLFFLSCCSTLLTWSLKSILSNTRKFSVEIWLPFDPYENTI

VYYFLHLYIFLSATNTVFCNSALDTLITGLIYQAAIQVRIIKSTIANLQAIADEKLLKPD

GILSFELRKNSNKKNNVIYEEITLCVRRYGQVKRFVTDIEDVYSFVLFSQFAISVLLICF

QCLQLSIVEPFSYRFFYIITTLITTTSEVFMYCYAGSLIYNETLSLNSDLYVSNWFDLDL

KCQRALIIIMEYGKHPFIIRAGKLVDLSLDTFTLIIRRSYSLLAILQNYN

>RpalOR38-like

MSIYPRSSHLKIPMIYSSLLGIFPVDLMFEGYPFFRKLYAKYSIIMIIYYVFYVITAYMK

LYELVIEPDPRLDEISRNLCITLIYTITVIRQVIMKVDKGIRNIIKTIIDNEEYIQGCED

EKIKKIFNDCSNDTTSKCTAYLIILFIITMLYIVRPLFVEGFIQVVGNSTTVIKSLPLSS

WFPFDEQVFYMPAYIWHIFDSLIGASFVTYTDILMFSIIVYPTGQLRVLIHVVGNFDSYK

QRTKKLYKIKSDDEAAFITFRECIFKHKEIISYVDNFNRTMSTLMVFDFLQSSLQIATIL

TQILGNDVTLILVLFVGAYFIGMILRLVLYYYYANDVVLLSAHLSVSLWQSNWYEQSSQV

QFMMAMFMMRCQKSLKLFIGPFSVMSLDSLISILKATYSYIMLMYGVN

>RpalOR69

MILSLLPCAIPIFVDFIYLFQDSIKNLTQIVENMIALNCIIGMIYMVICFISNRRTIQKL

VMSIYRFKKYGYWLKTIEVDERANLFCKVFMFYGILGNFVYMLMPQLSVQKCKASMVENK

MPCGLVVRSQFPFRFDHSPIYEIVFVHQIYTCTMVSIVVLSLTMLLCGLLMHTVNQLNNL

RAYIRKLQECQPDKLLERLIYVIRYHIDIIEYSKEIAGAFSTMLLFYITLTSLVLSVLCF

EIIMVDAFEDSVRFSLHLVGWLVILLSVCYNGQLLIDESLEVANDIYSIQWFQFSVDMQQ

KVQMVILRSQKPLSMSAANMGIVSLQAFLKVMSTAYS

>RpalOR13

MSLVSIPKICMLMIGVWPPELIENPVKRRFYLIHKYILITYVSSVTLSLTIEWIRLILYH

KAAGRVSSSGSLVVTIILLSFKLLIYQKNKVPHMFLSLLEFEYDLKSSDDQEVKQFYRKI

LKNNKYLCIGVIIATFFTISSFVILHLVKLWEIGVDDWDLNEAGFMYELYLPFDMKKYII

FVLFYNLYTATMGGVLNIMCLITMYTLIVFSALQLSILCLRIKKFHTFVGIYGNDIASTL

KVIIRQHQKSIEFVDNLNKSVRYVVFIDFLLNSANVASVLFYLVTVKSMYLFYAILLLTL

QLLQVFALAWVSNEVKLESVRVADAVYESPWYGQDQTKKLLHIILMRSQKPLAITIGPFK

PMTAETAIMTLQAAYSYATLMMNSYKDD

>RpalOR40

MEEQHFEDKLGTEMFKVAKKFMLLLGSWRINNANTSILLLYRIYYFICTSIFMFASILST

VKLMDLLYKANEMNLVVDQVGKVTLAIILIIKTRIYGGERMADIIQTGIRDTNEAIKLNL

DIRNIVLETVTYCNRTNFIIAGLTIVAGSLYNLNIIIEAYHFEKRYNFSMRPAYLLASYY

PYENYFYLVFTGEICLATYIILLTCSYQTLLISIMNNAVAQIRIIHYYFKCFGNNKVTEL

TENLLQKKLKNILIKHQKLISDMDYLNESINIAIFIEYVVSSLMIASMILQFINGVHISY

YAAMLVQVSFQLLLFGYCADEIKSLSEELSTTIYSTNWYKQSTTIKRLLFIAMMRSNKPI

VLTIGPFGAMSKESVIAIFKMAYSYVSLLSRGK

>RpalOR21

ELIELYKKHIKFLKYISQYRTCPYISSVGLVILYGGLGMLTYSAACLMLLNLFQGLLIAS

VTGLIIIFYSWIIMNAAQILENEFESIYNALYNVPWYNWNNSNKTKYLLILTKMQQPVQL

CLAFNIIINREYLTKYIKLIYICITFLYNFR

>RpalOR56

MFKKTQSTCCELSLKFFTFYQILPKDNEDTRSTKYHNFKIYFVLACSSAVIMIGCNMHSY

YVISNNIDINIDIDIAMSVSMLGIMWVNSLFVKNQHIYLEMIRSFSDFQTFKKPPGYDEF

NEKINFYTKLHLIYIHVGGMSYFTIFSHFRSKKCERINEERGLNEICLLFSNVYMPFDIN

FFPVVEIIAALQLFSSVYVYFLCGSILWTNVELVEHMGFRMRHLKCLLLEAFEEQHLKIQ

RRRFNYAFRYHIELLRIGKLLDKFYGIELILHVTLTGMTLGCCAFAMLNNNSLDTLILFV

GWLFAIVMSCIAGQRLLDETSALPDILYETNWYDLDVKLQKDLVLFLCRSNIPMEIKAGT

VIMRSSLIIQILKTAYSCLTLLSSVK

>RpalOR3

MFFDRGLFPTCKFMLALCGLGLYGNTNKVHQFLYTIFTIISQIIYCQCVVSFSVSAYLLG

FDEIIEALEGLSRLTYVSLNVAKMVLIQSKRMRNILIMLSLEEENCLKSNPNLKKIYIFH

VKYCYIMIFFVLIFLYAAGFINIEMGFMRYFEWKHMPNRNSSIPKPHTIPLWYPFDKEKH

YYIAVSYQIYHILQTLLISGSIQALMNSILVFIRAQLKMLQYEIKWFDWNASEQKYEYGD

FPRRNLRRLVIKHQEIIEWVHKFNGCFKTIFLLEYCITSLQLATTLIQIVQGSKLIFNLA

FFTHCSLQLLALSWNANEILLESSVGFNRALFECPWFRHDKYCQYIVIMMLLRCNVPLTM

TIGPFGEMSIDMAISRLKLSYTCLSILRATTD

>RpalOR39

MAPRTELFKVPRAALLIGGVMPSELLGWPTFHKQIYRIVSRLFQFIYSLCVVLFGTEIFR

LTGTNTRAAAASLKCGTFATVIFAKLVLIQSDKMKALLAKATREEKRINGTDEKNILRMY

GECVRYSNLTSYVTMAVITFSGLFFIIEGLTENNSDLPNTNNSLSLKRHPVQIWFPFDKN

KYYGLALSYEIYHIVGTVLYYSITQPLVVSVMVFMKAELKLLQHFLRNIRHDNTQSTQES

LIYVVKKHQNIINFIEDFNDSFQYIVLLEYCVTSLILAAILIDLIQGVKMQFNLSFFMIS

LFQLYSLSWNANEIILESSAGLSMAILDTDWYDMDKECAGIIRMMLLRCSKPLSISILGI

GQLTIDAAISRTKLTYSVFSVLSTS

>RpalOR1

MEKRVFSKDTEKNTRLIGFLKTLMICTGTWSYHWTDNFMLEKIYKYFSFFPLAVFAAFYF

LLMMELLRVLIEQDKLDSFLLNLGVFLDASKITIRFVFYFKNNVLRKFKDIMMDEQDIFE

SQEPEVIQYYLKRAKSWRSTLIFLYINTCLCSYGYMLVEISDNILVNKKHKINNESVEPP

FLYQIYFPNPDQNIIYIYWVNCFMVTFIILAVNVSFAISTTCMVYGATQLEIFQFRLKHI

HEVANKSYDGNIALTLKIYIQKHQKLISFITDLNDCIRSVTLGEFLISSINCASVVADIT

KVNDIGLSATYAGAYVLLLVAQIFTYASVASEIKIQSAAIADAIYECEWYNFDKSSQKMI

NILQARAQKPLEMTIGPFGAMTNETAVMMMKACYSYVAFMKDSGVANTSTE

>RpalOR55

MPTQNFYPFDASKQYIFWIVYIIESNYCFHICYAFSLATSIVTGLLMQVIAQLKHCGKSF

EDVFNSTPYTEDKEQVIKRRFQSCVRYHQAILSYSERIFNVFSTMLIIYVTITSFTLAVI

SYQIASSKTNVQDRLRYAMLLFGWLLLFSFICFYGQKIKDESMNIADAIYNSNYFEHDTS

LGRDIILIIARAQKPLTLKAKYMGTISLVRFVSVLKTAYSFFTLLLTVTDEK

>RpalOR19

MEVQFGDQMFSLCMKLLVAVGSWNITENMNPRIIPFYKIYTYFTKVFLFVVSILVTIELY

LQLFVYNKVENGVQVATILIILIIIYLKLRIITKENVLSLIRGSIQVTNTYTNMDSDIKN

IVQEQLKACSYANWPIVFICSSATLSFVIFRIFQVKQFKRENRFEDFVYYMYDLFDNFTV

VFYMENGVAMIVSVTNCALQTLLNTIIINLVTQIKVLQYQFSTYDVNADPYDDQKEYKLE

IKLKNLIRKHQNIINFIDKLNEAFANVVFVEYSISSVTIAFIVITINEENAYFYSLVLIL

TTVQLYTFCTNADDITTESSDIGTAIYSSKWYERSHSIKKMVLILMLRSKLPLALNIGKF

GQMSARSMISICKAAHSYLSFFH

>RpalOR36

MTTTVTTTIPERVDILVLVQKILKIFFQIPADRLSRTANNLVTLLGHFACFVNASGTVVA

VVYQFLIGIEDPYIIWETMIGVSDYLGFVYVSVCFRLKLKQLRRVLRGIDVFLDYCDVSV

IRAAEADVNFYTKCFVGYFLIGLFLNGLVPILGRHSCNERRVSDFYRKHDPCGMPVRNLY

PFDATDPLVFTVVYAVEAFTCFLVCFGILGITMTLIGFLIHIIAQITNCKKHILEIFEDN

EDPDYNKEKVHEKMAFCINYHRTIISYTESVYSIFNLVLIIHISVTSLIFGVTLYLITQV

ETYADKSRYSLQLAGWIALLFITCYYGQRIITESVTIADAAYQSLWYNGPVNLQKNVKLM

IMRGQRPLKLNVASLGVISLETFVGVMKTAYSFFALLITISD

>RpalOR44

MLAYNYKQYYKVDFEMLKYFGIWIEKNLTVSKRWYSIFININYIVISLVLIMDWTINTRS

VVDFGVTYIKPICFMAPIKSFLLLSNLNQLRSLLVDLEEPLFQPENRLELELVKKVLDNF

QKKKRIMIGIVGSGIVGSGCYPLFDRTRLLPFPAWYPFDVTDSLNFIFVYLHQFICVLII

GGMNMYCDIVITGMATFVGLQCQVLCLRLENLSKIAQDKRQKMFAKCVQHHVLLLRFLGK

VKYIFGIIYFWQICLCTMTLCMGLFLLSLSEPNSVEFFYLILYQIAISFLLLVPCWFATE

MTEKSEQIAIAAYFSNWVDSNDELKKDISFFILRSQDPLKLYAANFFEISVVTFVKIIRS

SLSYYAALNNLSSEEE

>RpalOR8a

SSIMDSLEDVIYDPKKNEAFYFSLAFLRVFLFYPKGSKQDRSKYYQVAAIFLRVVSTFVC

WECFLHIFMSIKNGPYVNFADDMVGVAGVANCFMCGAIFHYSNKKWSKFFIDLTDTVKFG

TPPGLNKVVLQNNHYSLFYGIYCYFGCILYSIVSLLETGACEQLNLEKNVHEICGTMSPL

WWPNDEINPWTKGIVLFYQLCCILVILPACAILMFLPLESSRLLSVKFRHLKQLIYQLFE

SEDDGNKIKRMAYCIRYHQELLR

>RpalOR28

MFRLLEDQRKLLSFTAIWPKSKPSIMYNIILLVNLLTAFLLCLSMMINTIYNSYDIVKLS

ESFVIFITVISSQIKIVLLVYNKKIFLKMLNNLNTENFSNCLWKYSSILENACQFYRNLA

YFYKIAVFICIVAYSIFPILENRKLPLDFPFFNDGPFHYPFYVFEMVSLSLAAFNNMSLD

LLILGVIYIAAVQLTILNQKLKDTDETVKKRTNSHSEIETLTMLYLNNCCQHYVDIERYI

NLITELLSIHISVHLGGNVVALFNSALSLMELSLFSIQGLTIISYIVIILFQSAIYCWFG

NLIYTESMEINNSCYTIEWYNRGAAVRKTMLILMERAKKPFEIKGCKYITLSLDTLINII

KWSYSGFTVLRSTMQDS

>RpalOR23

MMDFMKQELSVDERFLNVVKPFLIFCGLWDGDLIQSQRKRKIYRYYSWFLLFQYSLWFLL

MLIDFTSTVQDAERMASSGAIFISYCEMLTKFIIYRKNKVTLFFGTIKKYEQDIWSSNNP

KIIKSYTRQVSNCKKINWCSIIVTTVVCCSFLFGGVLDDQENKRFGKAENYRPYRKMYQV

WLPFDVRDRESVVLVINLFIEIPGMVLYNSILMILVTFSLFLATQLKVLQIKLSEITNDP

DRIDLNQHVKELIKEHKYIIRETKEFSHAVRYIMLMEFLLNSVAVAAVVIQLLTAQTFEE

YTFLVSFAATLYLHIFSLGWTANEVKIQSVNIADAVNEMNWYNGDLDFKRMIHMVMLRAQ

EPLIIEMGPFAAMTIDSAIVSMKAAYSYLMVMKSF

>RpalOR11a

MYEPQKDDMFYMTLKFLRFSYLYPFNSDIEHQKRYYFLSCLLRFISSFVLFECIVHTTMS

VINKNYFILTDDIVGLTGLTNSMILCITFQLTVKEWSQLFKDLTNTSKFGEPKQLKQVAI

KINLYSSLYSGYCLVGLSIYTVVKLLETGYCEELNRERNLHEVCGTIAPFWWSSNEISPI

IKFLIVFYQMYCIGAILPPSAILCFIPWECAKILSVKTGHLKALFLKVFDSDNAAERKRR

LRFCVSYHKEILRLAGQINAQTKRNIGQLSFVAGIVMSCIGNQMLKQYSIGALLHLTGCF

VAIYLVCQAGETIKNDTESICDSIWFSNWYMDLELAKDLKIVIQRSQKPVTLQSYLLGDF

DNKLLIVIIKTTYSYLTLLTRTSEIE

>RpalOR57

MQNFDWTKTLNYERNLLKFSGFYTELKTRENLYYYMRKIFFSMMTILFELSITISVFYAC

DLIHLTKSMIFFILETSLVLKFLILLWHFQELNIAEEILNEFNNYQINNSEAKQVKESLK

SMMSFTKLYRRNCLFTSCMFAIPPLLVSGKYTLPTSIYLPYEITNNMTYIISFVYEILLV

VVSAHLNTLIDILYIKLVSLGSCFFEVLIHRLNEIGCFVEQEANQHFRNCLLFHEKTLRF

ISVIEKIYCYITFSQVAGSAAVICFSALGILVVPIGSGDFGLNLIFFINMTAQVALYCWY

GHNMRDLSIELNNACYMSNWYNTNRSIKKMLIIFMERSK

>RpalOR59a

MYSMYNTLLYSLALIFLILEIIAFTSFFGESQMIVRQISMMVTHLLGLAKFLVLATQHEA

IAKTQNRLQDKTFQYEPFGKFQPGLTMQEQKRISGIICTFLFCFYSFVGIIAHISAVILV

KKNTINDIFIMNTTCGSILPYSFYFPFDVSTKDKCYYSLFFMDVCLVIFAFYIATLDAVF

VSLLHLLKTQLNVLTDALSNIRGRCLNKLGLNEHFHILNDQEHPQLEDEMYKNLTHCTKH

VQSLVEIGSDIENLFTIVTLMQTLSSLFIFASSLFSVVKEPKSSPNFFSQLQYFVAVLCQ

FSVYCWFGNQITLAADRIPYALYSNDWFSCSKRYKNLMLMTMTRMQKPLYISIGKFSPLT

LTTLLAVIKASFSYFTLFQSVGTSG

>RpalOR51

MTEIFRHTTFIQQEIHRLRILGFYPNDSKWKFFTYLWTIVVYLITFAQMWTVIALTIHSP

DLTVISEIFLFSMTQVSFVNKLVSFHLKLDNAAELERMLSRNVFTRVTPAELGVMKAEFR

KCQGVLNVFIVLCFGVVVFYTVFPAIDQGQPGKKNYPFPGEFPFNPDDYYVWIYCGQILT

VASSAWTNSAIDCLFQKHVTMATVFFQILCERLRNAMGKTNEPNDSISRKIKKCVIYYNA

TTEYVSMIQRTFSYGILLQFLCSVIIICLTVFQVSLVVEYSSTKFVLMIIYLLCMLFQVA

LYCWYGHMLMEKSNEITAACYAITWNDMSVHNQKLLITVMERAKRPVELNASGVFKLNLS

TMVTILRTSYSYFAVLQQIYRK

>RpalOR51a

SVAVICLTGFQMLIIEIESVQFSLLLVYFSCMMCQVAIYCWYGQLLMEKSDGVTGACYKI

NWHRASTTNQKMLITIMERVKNPVKMRAFGFFELNLATLMKILKNSYSYFAVLQRLYTTN

N

>RpalOR66

MGAVKVCFWYFKSSCMRTIINTLEDPYFRYENYLNFQPGTIWKKYKFIGIRYSILFLGLA

HATLISSYIPAISVAIKHIRFNGKNTTTELPKALPYYSWMPFQYNTGYTYLLAILYQGGP

MISYAYSVVGMDSLFMNLMNCVTANVQIIHGAFYTIKERCVNRVMKNKLYMNNLRESTEI

MNEMDTEMEKIIKHLQTLFKCCYELESMYSFVTLCQVTATLFILCTCLYLVSMADPFSKQ

FLAECVYMAAMFFQLYLYCSFGNEVTLKFQEIPFHLWSSSWLATSTSFKKRMMFIMLRVK

QPVYFTVGKFTPLTLSTFMSIIKTSYSIFALIKNTSP

>RpalOR15

MLRGDKLFRIAKLLMLIAGTWRLELKELSKKWQMVYKVYSVFIQISFNTFVISFLIELCI

TWGVDNDKAVECLTTLIVNFVFVVKMGLFQTKKMTKLLNRIIDHVNSLSINDRYGEDIFN

SHLRYNNILNIGILVYTFSLGLHLNIIGLVQYVYFQKETHNSTITADKPIILMYWYPFDY

NKHYGFVIFYQLFTILASDLYNSAIQALFNTIIIQLSSDLKIIQLEFQRFTLNSGLSKQN

LKNIIMKHQKIIKYIEDLNETLKYPLLMEYTVSSVMFASVLVQILQGKKIVFNAEYFVIM

AFQLFILSWNANEIQIQSEQIAYSIYESIWYEETTDVKKMLLIVILRCRKPLSLNIGPFG

SLTNNAFLSRIKLAYSYVSLLSGGANT

>RpalOR11b

MYEPKKDDIFYLPLIYLRFTFMYPFDSDLEHLGRYYFLMGLTRLTSSFILLECILHTTMS

IINHEIDFFILIDDIVGLTGMINSMTVCIMFQFTVKKWSRLFKDLTNTSKFGEPKQFRQA

AIKMKLYSVLYSAYYVVAITVYSLVKALDTGYCEQLNKEKDFHEVCGTITPFWWPYKDIH

PIIKYFVIFYQLYCIGVYIPTSGMLCFILWECAKILSVKIQHLKDLLLTVFESENPARRE

KRLHFCIAYHCEILRLAQKTNERTQETVGHLSFLVAIVMSCIGNQFLEQKSLGSVVYLGG

YLLGISMVCHSGQMIKDETESISNQVWYLNWYKDLKLAKDLKIIFQRCQKSVPLRSHLLG

DFDYGLFVVIIKTTYSYLTLTYDKKQN

>RpalOR4

MKHEKLIELARWHMCVGLWNFKKSNPYRKHLYQIYDIYKALLPTYMLLFIISFIISTIQM

IRNKEPIADISLSVAFLLSNLDISSKILIFKKNRVPDWLYLVSDMERNLWKHGSEEMKKY

YMKQVNLCKIFVLISTIMIIFSMSIFIISKLQELFLNEIAPEKGLLPMWLPFDKSEHAVF

AWTMEIWICYFNVSLFVTVKITIVTLIIFLYTHIRVLQMQVKSIGSQESHEDDVITYIKQ

HQHLISLETRLNDILKYLLLQEYFLNAANIAASLVQILVLKSFLEVFYMVLHCLYASFQI

FALGWVANEVKEESLGISDAVGNSFFYKCKLSTQKSLLIMMMRAQRPLMLTLGPFGPMSL

STSISVVKAAYSYATLTLNRYNAN

>RpalOR17

MLRPMQVFGLYPTDVLPKWVYYLHAGFLHCTLTLSVPIFVLIYLFVNNDLSARVVMDSLF

MFSETGITVFKYATIRLWPEYTRRTLRWLENETFNTFYDDHKEIVLQTSKSCKNTYLYFV

ITCVITWLTWGLRPLLYEKRTLPTPIWLPYDPYANSFIYYMSMSFIVLSVGNACFGNSST

DCMVAGLVSQAAGQIKLIKAVLRDVIKHADEYVTGFSSNLNKDELISLRNQHIYDDICRC

VDRYNLIKDFVNDIEDTFSMVLFWQFGASVFVICLGFVQFLFVELWTFDFFAIISFVTTM

SWEVFLYCYYGSLLSNESDTLVTDIYTTDWLDLELRCQKAYLTIMENGKKPLILKAGKII

DLSLDTFATIMRRSYSVLAVINEVIE

>RpalOR54

MSKIHLLRQARILMIIGGVWRIPFSKNPNVYKIYKFYAYIIQLFYVLVACCMFTEFFYLL

NSEDVKKLTDNLKITISCSLVAVKALIFQAGAFPKLIKNMIMDEKNILENTNDEIKTLYW

QFVSYINKCATCILGIAGITAFLLLMAGSVIGLTSKSETKPMLFLAHFPFDQQKYYTASM

IIQMIAVVLATMYFCMSQIFYLCILTFVKAKLKILQYHFKSFKNEPGKRAIYIIKSFIKM

HQNIISFVDDLNASIKYLLLIEFLLSSVNIASVTYQLISSLTIADMMFSFSYLMLLVGQL

FILAWHANEIKIESVAVSDSIFKSYWYETDVDIQKLIFMIILRSHRPLLLTIGPFKPLTT

HTVISVMNAAYSFIMIMTNSN

>RpalORu2

MFASMCYLVMECHMAQFKSIIILLNKETCNKIFAMIDHHYFQPRNLHEMKIVKEVLDKFS

ILKKWMSSISMGAVIMITIYPMYNGEHLTPIRSWYPFNINYSPIFELFYAHQTFAIWSIG

IINIYLEVVITGFLTFIGLQFDLLSFRSENIQKFTTSEIKQIVWNHHQILELFALVKGVF

GKIYFWQIFTTTITFCMNMYLLSVADVRSFEFIYLFLYQTAIFLLLLFPCWFASVMTEKS

EKVPVALYSCNWFDASEDFKKQLIYVMMRTQTPLMMLANDFFYISVELFMKIVKTSMSYF

AVLTNIDASENQLSI

>RpalORu24

MIVFMCSPIIMEFLHVITGGSGDFSRDILTFSILILHSMGVLRVLGYLPVKQNYEDIITF

IRRPSFNFNVFRYSSLSMIQRNNDKELISRHPIDEFKDTIYKEMDHFCIKSLIVYVSYVQ

INLISSYGQNYFNQPTYEQFNPRLNRTSIYRDYVYFLWHPFDISISDGYYLLGFFYQPVS

FSLLMSSFLCIYMLDTSFILYLKGQADILNYAFSHIDKNIDESMNYMDIIKLKEIRIVKC

INELQAIYHCSSMINNLAAFPMLMQIFFCSALSCGLMYRIEYVDTLVEYLYLSSTVGICW

LIIFIMCWYYQEVTLSIMDIQFKVNELDWLNYSNKLRTTLLFTIARLQNPFHFNMGHWTP

INTGTFIGMIKMCYSAYMLVKSTAENEAT

>RpalOR35

MLKLVQIPGQLLKTVALWPLERPSTTYKLRTTFTFLIIVLLAMCMSINALYHMNDFVQLS

KSLYLLISVSLSIMKTSVIFWKNDTLFAILEHLDKPSFTSCLSQYGDYPKKAYNVFKILT

PVYAGHVVVFLILMCVFPSLEGKRLPLDFPFFNYGLLYHPFYVFQVLATAASAHANLSVD

ILTVVLISISTVQLRILNNKLVDTDDNISALEPTSDKNIELLTVKYLNNCCIHFSDIENF

VSLTEDLVSVIIFFQLGASVVAICNSCIMILSVDAFSTESIAIIIYLNSMFAELGMYCYF

GNEVYLQSLEIHQSCYFSKWYDRGSSVKKILFILMERTKRPLQMRGCNFIALSATTFVSI

LKWSYSYFTLLRNTMQEAN

>RpalOR53

MINGIEDVNVLGESFIHFLNYWGYVYVYYCNKRSYESTKKLLKDFDIFKIFCDESISRNV

ENKVTKYAKGLLWYGLVGITFNALWPIITKKSCLSARSESYRKHDPCGLPTHHWFPFDAS

TFAIYCTIYIIENFAIVVAIMGFVFLPMTIGGIYSCSFLEVFLKKKTISKLYRQF

>RpalOR8b

VILPACAILMFLPLESSRLLSVKFRHLKQLIYQLFESEDDGNKIKRMAYCIRYHQELLRL

AGELSRLTKRMFGHLSIIGSLALSTIGYQMLENSSMGAFMHLAAYMVGIFFQCAGGQILK

DETSDFSTDVYMATWYDDVKLAKVLKFILQRSQKSVALESYMFGDVDNQLFVMIIKTAYS

YLTLLTTFS

>RpalOR62-like

LLAGIWKDRIRDRKWLQRLYVTFWAMLQLYYAYMVAGTAVYLKHTLQQWDMQTLTNHVGH

LVVTSMIWLKVLLCQSKSMTRMVSKCEQEERRIYASESARLKENYEDYVKVTTRLSS

>RpalOR27

MFDLCRRLLILVGSWNIMDNMSPKYLRFYKVYIYFARTFLFAIFLLVSIELFRQLYIRQD

FERGFQLASTHILIAIIYLKLRIYKNKNVLKLLREGIRVTNTFSRMNINIKNIVYQQLSS

CNSANRPIVAISISTGLCFNIFRIFQAKQFQNENRIEDFINFIYNPFSDLSVFVYVENWV

AVFFSVLNCALQTLLNTIIINLVTQIKVLQYQFSTYDVGESCDYTNEKKLFRKLRALIKY

HQTLISYIDELNESISNAVLIEYSISSLTITFTVINISKHEDIYFYGLVLMLTTVQLYTF

CTNADDVIIESLELGIAIYKSAWYERRQPIKKIVLIIMMRTKLPLALRIGKFGQLNARSM

ISICKAAHSYLSLFHGSN

>RpalOR64

MEITLGEKLFKLSKNLMILVGSWQLTDDMSSSYLLNYTIYSYFSFFILAGITVSGTTHFF

VEMLSYRDYDKGIELMGLNITLIVMYIKLRTYKSVTVTSLIKELIRMTNESDIDDNARDI

VRKRIKYCDNSNITIFVLSVAVSTYYGINGILEFHKYEKESDGTTTPNHLLFMYYPFPNY

IHFINYMEKIVGVYLGFYNSACLTLLNTIITAVVIQLKVLQYEFSNYDSNSKTKSSSPIL

KLNGLIRKHQHLIRHSSTLKKAIEIPILIEYVISSLSIATILLQILKGSDVLFYIFMVIL

ITIELLFFCLNAHNIIVESLGLEEAVYFSNWYERDLSTQNSIRIIMMRSKIPMHLSIGPF

GMLNAESLIKICKVAYSYVSLLSREK

>RpalOR63

MDDEYKEFHMSLTRKYMTLLGIWPVNWTGTRLILYHIYYYLIFGYFCFLVATMYVQLGIS

FSENVLAVAYAAGPTVAYSMNILKICVYKTDKFLNIINEIQKTEKKILSSENNTVIDIYK

RNASISKKIEIYLSLMGVSGIAAYFTGPTLVDRNTSQRSTLSKNLIIPSWYPFDIQTYHW

VAYGFQVLPCFYGTLYIVYTQAYYLSILIFIRGQFKILQFTIHNTFSGNDTHRNLGNWIK

EHQHILRMMVALNERMSIVMFFDFMVSSLQLAMVIFQLMNVSGLEQLAVALFCCTLNIQL

YLLYWCGTEIAFESGNIAVSAYQSNWYHCDVTVQRALQVIIMRAQKPVMLKIGAIGTVQF

DVLLKIYKTIYSFLCLIYK

>RpalOR17-like

MDCLVAGLICQAAGQIKLIKATLRDIIKHADERLTKHSSKLSADELTRLRNQYMYEDICK

CVDRYNLVKDFVNDIEDTFSAALFWQFGASVFVICLGCVQFLFVELWTFNFFSIISFVIT

MSWEVFLYCYYGSLLSNESDTLITDMYITNWFDLDQKCQKAYLTIMENGKKPLVIKAGKV

VDLSLDTFATIMRRSYSVLAVISKMIDN

>RpalOR50

MESQSYFDWGDIFSLHIKVFIYCGFWKFDTKSTWTKLYQLYTCFGYLLMFSYILSQIMYM

IFNRNDVLEVTSAMYVTCTFSTTFIQGLMVYYNLDTIKELIENLRTNELLQVKCKHHLVV

VRNTKQILRFFYISSIVLASGTQTFWSIKPYIKGTHILPTKAWYPFNYTTSPYFHITSTY

QALTTGYMIIHTLNMDLVIDIFIIILGGQCTMLKYTLENLNHFEVINGSLIERHTKIEKD

NEGFIRVMTENMKICIQTYNEIKILSKTFEDIFHIGLFIVTLGGGIILCSILFQLLYVDM

GSNEFFYLIFYLFSMLLQQSVFCFLGSQLTTKSEVLYETILNIPYWTDCGINFQKMMIMF

LIYVEKPIIIYIGRILPLSVVVIVNVLRTAYSYFTLLVNMQSVKN

>RpalOR67

VKSAGELFYLTSMAGVSLLICFFMCWYGQEFTLVMDISNRMQDLDWLSYPVNIQRTLCFL

MMRLQKPFWFRLADWTPSDTGAVFVLVKTSYSFYTLIKNTGSNIKT

>RpalOR32

MAIFDLKVDLFGRCVELYYLMGIIPLLPDKRNKQGLGYRIYSFAMLYSGYICTGLEFVNL

VQSILSRNTSLEIIITNYMLASLHTAGLLKSRLGNSKKGAEYLQKIIDFEKKVYASRSKD

LINLYKRNGAAFKMTAAGYFTGVFMVVVCYGSSSLFRSQEFVLIGNVSYEIKQLPIPIWS

PFNQYTQYLLTYLWSLVIATELAFYFVAGDLTCFGYTIFALCRLEMLKYFIIEFFTKAKE

IQQNLKCDEIESYRILQRECIIMHQDIISYIDDLNSQIKYLMLMDVLPGSIQIAALLYQL

MNNLNVVQMVLIGEFLLTLTARFFIYCLNANKINEESQLIGTVWYQIDWTVLPEDVKRNI

VLCIKRTQKPLSITVGDFQEISLVTFVSIVKGAYTYMMFLTTF

>RpalOR10

MLVVLALIHFLFCASRYSTYINNKDLNNTIEKPLPVPQWHFFNTNNHYTLVYLLGWAVSI

IECTYSLLTITLFLTLMIFVLSQIKELHIIARDVDFYSVTLKMDHLHVLYVLVEKHQNII

RYVTELNKSMRQILLVEFLLNSINIASVTVVILVFEMPVVYLLFNINFVALLVVQLFFLS

WHANEIEIQSLLLCESLYNSEWYNFEAEAKKVINIMMIRSLKPLNIYIGIFYPMTLGTAL

TTMKTAYSYVTMIISFTDN

>RpalOR31

MYEIRKDKPFYYSLRLLKTLRFYPQDYDGDYKTRFFVSFVIWNIIFGVPWIALLLAHLIV

NLVNRTEADLSEDIGDIFGDVGAMLCCIFMSIHYEEWSIMFQRLSDFKNFGKPPAYEQAQ

RRGNLISKVTFLYGILGISMYVGLSMMDSKRCHRLNAEKNLHEVCGTFSPAWWPNGDQIG

FWEHVFLLTLQIVSGMIYLTSSFVLNTIPLEGYGLIISMIDHLKQQIIEILQIENVDEQE

WKIKKMISYHQYIIQLSEDLNSICKVTIGHVYLIAALVIGCLGNQLLHQSTPKALAFIFA

YSVAMFYSCHAGQIISDESLSIGDAVYNSNWYEAKPKIRRYLVLMILRSQKPIKVDALPL

GTADYPFYLMT

>RpalOR25

MGMFNFLKVNLFGHCMELYYIMGMMPNLPDGSNKAGWIFSTYSFLLYYSSCVCNSFEFVN

LVQIFMDKKERNLEAAITNYMLASLHAAGLLKSGLSKSKIGAKYFQNVVNFERKVYESGN

KEVIDVYENNCKAFRATAARYFTSVFLVIVCYGSSSLIRSKELVVIGNLTYETRQVPLKI

WSPFNQYTQYFWAYLWAISIGTILAFYFVAGDLIFFGFTIFALCRLEILKYFIMNFYKKA

NEIRYHLNCDTVESYRILQRECIIMHQEIISYIEDLNSHIKHMMLMDFLPGSIQLAALLY

QLINNLNVIQMVLIGEFLLTLTARFFIYCLNANKINEQSQMIGTVWYQIDWTILPEDVKR

NIVICINRTQKPLAISVGDFQDISLVTFVSIMKGAYTYMMFLTTF

**Appendix S5. Comparison of RpalOR32 and ItypOR33 sequences and 3D structures.**

Sequence alignment and binding pocket prediction of RpalOR32 and ItypOR33. (A) Amino acid sequence alignment. Transmembrane helices are indicated by black boxes. Residues predicted to form the binding pockets are highlighted in orange (RpalOR32: E72, I73, T76, L80, L83, H84, V143, V146, V147, Y150, R157, Q173, L174, P175, I176, P177, A199, F206, Q299, N303, Q308, L311, I312, F315, L319) and purple (ItypOR33: I82, L85, H86, V145, I148, S149, Y152, S153, M178, G196, G199, V200, T203, F206, F315, T318, L319). (B) Predicted 3D structures of RpalOR32 and ItypOR33, with predicted pockets and transmembrane domains. All residues constructing the binding pocket are displayed as sticks and spheres.
